# Volatiles from the tropical ascomycete *Daldinia clavata* (Hypoxylaceae, Xylariales)

**DOI:** 10.3762/bjoc.14.9

**Published:** 2018-01-12

**Authors:** Tao Wang, Kathrin I Mohr, Marc Stadler, Jeroen S Dickschat

**Affiliations:** 1 Kekulé-Institut für Organische Chemie, Universität Bonn, Gerhard-Domagk-Straße 1, 53121 Bonn, Germany; 2 Abteilung für Mikrobielle Wirkstoffe, Helmholtz-Zentrum für Infektionsforschung, Inhoffenstraße 7, 38124 Braunschweig, Germany

**Keywords:** enantioselective synthesis, gas chromatography, mass spectrometry, natural products, volatiles

## Abstract

The volatiles from the fungus *Daldinia clavata* were collected by use of a closed-loop stripping apparatus and analysed by GC–MS. A few compounds were readily identified by comparison of measured to library mass spectra and of retention indices to published data, while for other compounds a synthesis of references was required. For one of the main compounds, 5-hydroxy-4,6-dimethyloctan-3-one, the relative and absolute configuration was determined by synthesis of all eight stereoisomers and gas chromatographic analysis using a homochiral stationary phase. Another identified new natural product is 6-nonyl-2*H*-pyran-2-one. The antimicrobial and cytotoxic effects of the synthetic volatiles are also reported.

## Introduction

**Scheme 1:**
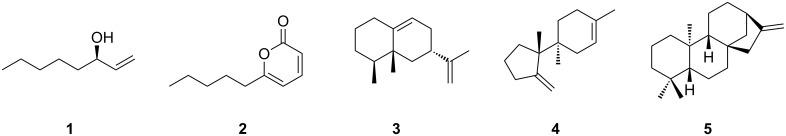
A selection of widespread fungal volatiles.

A large variety of volatile organic compounds from different compound classes including fatty acid derivatives and polyketides, aromatic compounds, terpenes, sulfur and nitrogen compounds, and halogenated compounds is produced by ascomycete fungi [1]. Possibly the most widespread volatile secondary metabolite from fungi is (*R*)-oct-1-en-3-ol (**1**, Scheme 1), a compound that was first isolated from *Tricholoma matsutake* and named “matsutake alcohol” [2–3]. This odourous volatile is responsible for the typical mushroom smell of many fungi and also contributes to the pleasant aroma of edible mushrooms such as the button mushroom, *Agaricus bisporus* [4]. Another widespread fungal volatile is 6-pentyl-2*H*-pyran-2-one (**2**) that was first isolated from *Trichoderma* and exhibits a strong coconut aroma [5]. For fungi producing **2** a plant-growth promoting effect and an induction of systemic resistance in plants has been observed which makes these fungi interesting as biocontrol agents [6]. On the contrary, fungi can also produce mycotoxins, which must be excluded for their safe usage in agricultural biocontrol. Some volatiles, especially terpenes, point to the production of certain toxins in fungi, e.g., aristolochene (**3**) is the precursor of PR toxin in *Penicillium roqueforti* [7–9], trichodiene (**4**) is the parent hydrocarbon of the trichothecene family of mycotoxins in various *Trichothecium* and *Fusarium* strains [10], and the diterpene *ent*-kaurene (**5**) is the precursor of gibberellins, a class of plant hormones that are produced in large amounts by the rice pathogen *Fusarium fujikuroi* and other fusaria [11–12]. The potential beneficial bioactivity and role in the intra- or interspecies communication as well as the possible function as markers for toxin production recently resulted in an increasing interest in volatile secondary metabolites in the scientific community.

The Xylariales (class Sordariomycetes) is one of the largest orders of Ascomycota and comprises several thousands of microscopic fungi, as well as numerous “macromycetes” that may produce conspicuous fruiting bodies (stromata) [13]. The Xylariaceae remain the largest family of this order, even though it was recently further divided, based on a multi-gene phylogeny that widely agreed with important chemotaxonomical and morphological traits [14]. A comprehensive overview of the current taxonomy of these families has been published by Daranagama et al. [15].

However, still only little is known about volatile secondary metabolites from Xylariales*.* Most respective studies have been dedicated to some endophytic strains that can be assigned to the Xylariales based on preliminary molecular phylogenetic data and are being referred to the suggested genus Muscodor. However, this genus was recently rejected, because its erection did not follow good taxonomic standards [14]. The only comparative study available on the production of volatiles that used taxonomically well-characterised Xylariales relied on a panel of strains of the genera *Daldinia* and *Hypoxylon* and some allied genera that were previously included in the Xylariaceae, but have recently been reassigned to the Hypoxylaceae [16]. Since many of the compounds observed during GC–MS analyses in the volatile profiles of these fungi could not be identified with confidence in the latter study, we have selected some of these strains for intensified evaluation. Here, we present the identification, synthesis and bioactivities of volatiles emitted by the rare tropical hypoxylaceous ascomycete *Daldinia clavata*, which has hitherto been only infrequently reported from Africa and Latin America.

## Results and Discussion

### Headspace analysis

The volatiles emitted by agar plate cultures of *Daldinia clavata* MUCL 47436 grown on YMG medium were collected on charcoal filter traps by application of a closed-loop stripping apparatus (CLSA) [17]. Dichloromethane extracts of the charged filters were analysed by GC–EIMS, followed by identification of the captured volatiles by comparison of the recorded mass spectra to data base spectra (NIST and Adams [18]) and of measured retention indices to reported data. For unknown compounds a structural proposal was developed by interpretation of the mass spectra, followed by the synthesis of reference compounds for unambiguous verification of the suggested structures.

#### Volatiles from *Daldinia clavata* identified by GC–MS

**Figure 1:**
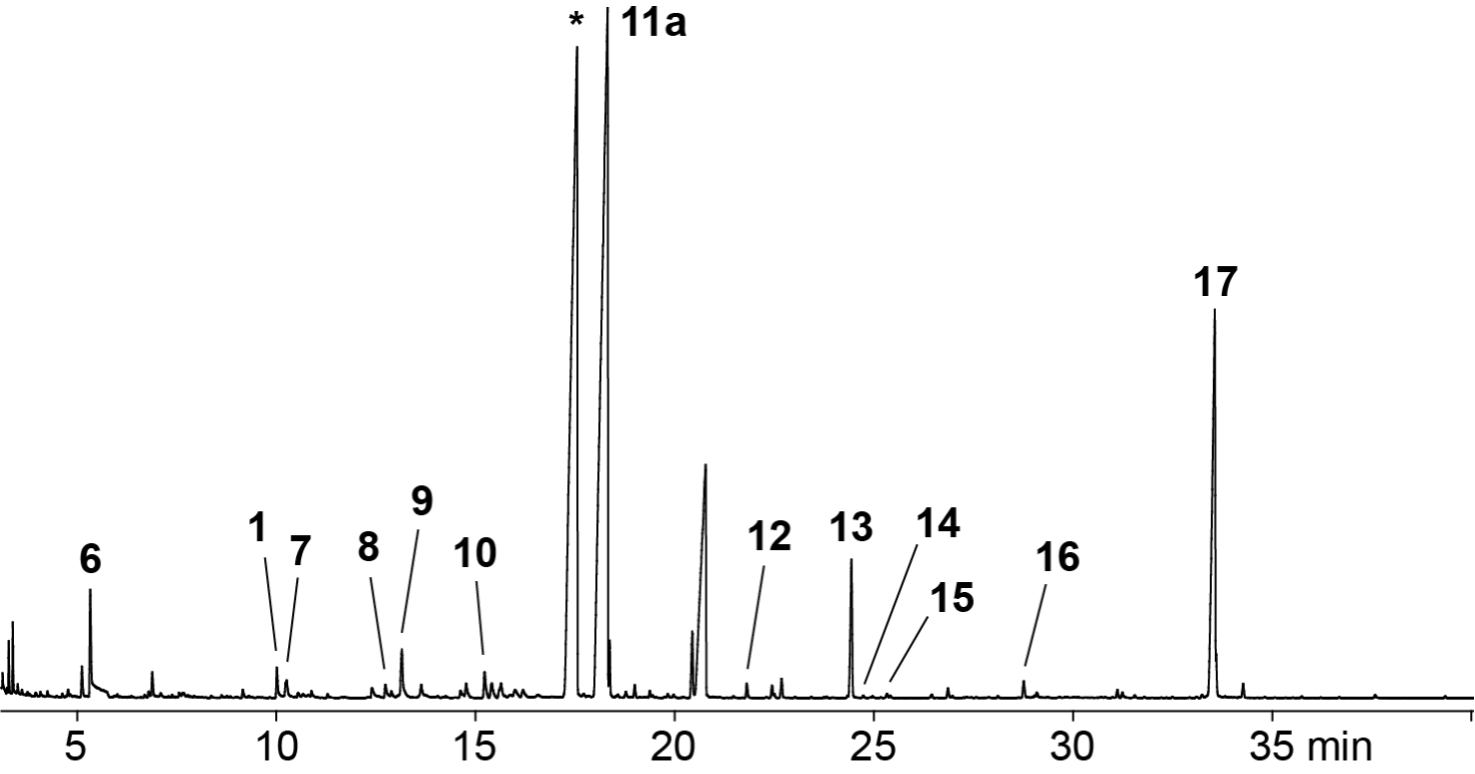
Total ion chromatogram of a representative headspace extract from *Daldinia clavata* MUCL 47436. Peak numbers refer to compound numbers in Scheme 2. The peak labelled with an asterisk represents an unidentified natural product from *D. clavata*.

**Scheme 2:**
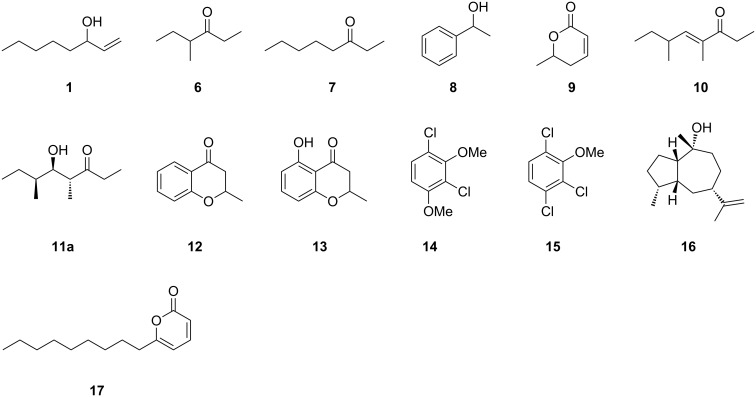
Identified volatiles from *Daldinia clavata* MUCL 47436.

A representative total ion chromatogram of a CLSA headspace extract from *D. clavata* is shown in Figure 1. Several of the emitted volatiles were readily identified from their mass spectra and retention indices, including 4-methylhexan-3-one (**6**), oct-1-en-3-ol (**1**), octan-3-one (**7**), 1-phenylethanol (**8**), and pogostol (**16**), which was further confirmed by comparison to authentic standards for **1**, **7** and **8** (Table 1 and Scheme 2). Furthermore, the two structurally and biosynthetically related compounds 2-methyl-4-chromanone (**12**) and 5-hydroxy-2-methyl-4-chromanone (**13**) were tentatively identified from their mass spectra. Compound **13**, which exhibits antimicrobial activity, was previously isolated from various species of *Daldinia* [16,19–22] and several endophytic fungi, the latter of which have only been tentatively characterised at the genus level [23–28]. The compound actually consitutes one of several chemotaxonomic marker metabolites for the clade in the Hypoxylaceae comprising *Daldinia* and allied genera [29]. The sesquiterpene alcohol **16** was first isolated from the plant *Pogostemon cablin* (patchouli) [30], but is also known from fungal sources [31–32]. We show here the corrected structure as reported by Amand et al. [31], while for the compound from patchouli oil the opposite absolute configuration has been assigned [33]. The absolute configuration of **16** from *Daldinia clavata* remains unknown.

**Table 1:** Volatiles from *Daldinia clavata* MUCL 47436.

Compound	*I*	*I* (Lit.)	Identification^a^	Peak area^b^

4-methylhexan-3-one (**6**)	845	842 [34]	ms, ri	2.0%
oct-1-en-3-ol (**1**)	981	974 [18]	ms, ri, std	0.4%
octan-3-one (**7**)	988	979 [18]	ms, ri, std	0.4%
1-phenylethanol (**8**)	1060	1057 [18]	ms, ri, std	0.2%
6-methyl-5,6-dihydro-2*H*-pyran-2-one (**9**)	1072	–	syn	0.9%
manicone (**10**)	1136	–	ms, syn	0.4%
(4*R*,5*R*,6*S*)-5-hydroxy-4,6-dimethyloctan-3-one (**11a**)	1228	–	syn	35.2%
2-methyl-4-chromanone (**12**)	1366	–	ms	0.2%
5-hydroxy-2-methyl-4-chromanone (**13**)	1467	–	ms	2.0%
1,3-dichloro-2,4-dimethoxybenzene (**14**)	1480	1487 [18]	ms, ri, std	0.03%
1,2,4-trichloro-3-methoxybenzene (**15**)	1504	–	ms, std	0.1%
pogostol (**16**)	1653	1651 [18]	ms, ri	0.3%
6-nonyl-2*H*-pyran-2-one (**17**)	1875	–	syn	9.7%

^a^Identification based on ms: identical mass spectrum, ri: identical retention index (standardised GC retention based on comparison to *n*-alkanes; for C*_n_*H_2_*_n_*_+2_ the retention index is defined as *I* = 100·n), std: comparison to a commercially available standard compound, syn: comparison to a synthetic standard. ^b^Peak area in % of total peak area. The sum is less than 100%, because compounds originating from the medium, unidentified compounds and contaminants such as plasticisers are not mentioned.

The headspace extracts from *D. clavata* also contained two chlorinated compounds as indicated by the isotope pattern of the molecular ions in the respective EI mass spectra. The first of these compounds was readily identified as 1,3-dichloro-2,4-dimethoxybenzene (**14**) by comparison to an authentic standard and to synthetic standards of all possible positional isomers. These isomers have been made accessible during our previous work that resulted in the identification of 1,5-dichloro-2,3-dimethoxybenzene as a headspace constituent of an endophytic *Geniculosporium* sp. [35]. The mass spectrum of the second compound pointed to the structure of 1,2,4-trichloro-3-methoxybenzene (**15**) which was confirmed by comparison to a commercially available reference. Chlorinated anisoles are well known as drinking water contaminants that can be sensed by humans with extremely low detection limits [36]. Their origin by biomethylation of the corresponding phenols is frequently discussed, but the de novo formation of compounds **14** and **15** without administration of the corresponding phenols has not been reported before.

#### Volatiles from *Daldinia clavata* identified by synthesis

**Figure 2:**
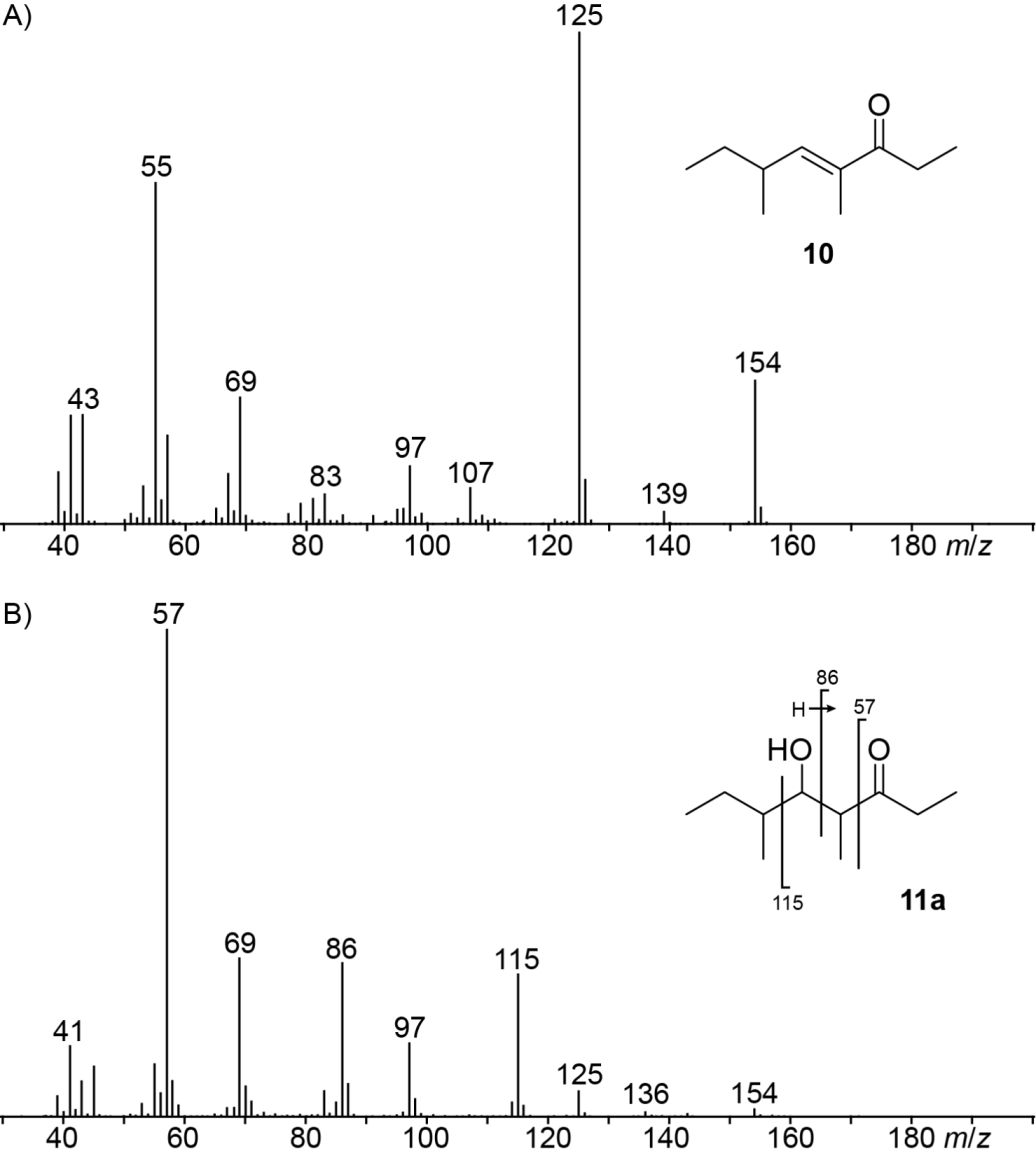
Mass spectra of volatiles from *D. clavata* that were identified by synthesis.

**Scheme 3:**
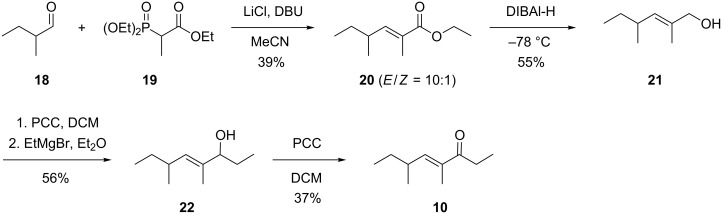
Synthesis of manicone (**10**).

Several other compounds released by *D. clavata* could not unambiguously be identified based on GC–MS data only. In these cases structural proposals were delineated from the recorded EI mass spectra and the suggested structures were proven by synthesis of a reference compound. The first compound showed a mass spectrum (Figure 2A) that was similar to a data base spectrum of manicone ((*E*)-4,6-dimethyloct-4-en-3-one, **10**), but no retention index for this compound was available from the literature. For unambiguous structural verification compound **10** was synthesised starting from 2-methylbutanal (**18**, Scheme 3). A Horner–Wadsworth–Emmons reaction with triethyl 2-phosphonopropionate (**19**) yielded ethyl (*E*)-2,4-dimethylhex-2-enoate (**20**) as a separable mixture of *E* and *Z* stereoisomers (*E*/*Z* = 10:1). The purified *E* diastereomer was reduced with DIBAl-H to the corresponding alcohol **21**. A PCC oxidation and addition of ethylmagnesium bromide gave **22** that was subsequently oxidised with PCC to the target compound **10**. Comparison of the natural product to synthetic **10** established their identity by same retention time and mass spectrum.

**Scheme 4:**
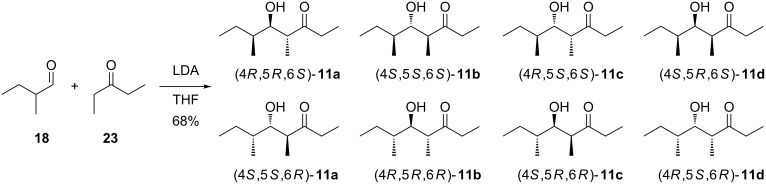
Synthesis of a racemic mixture of all four diastereomers of **11**.

**Figure 3:**
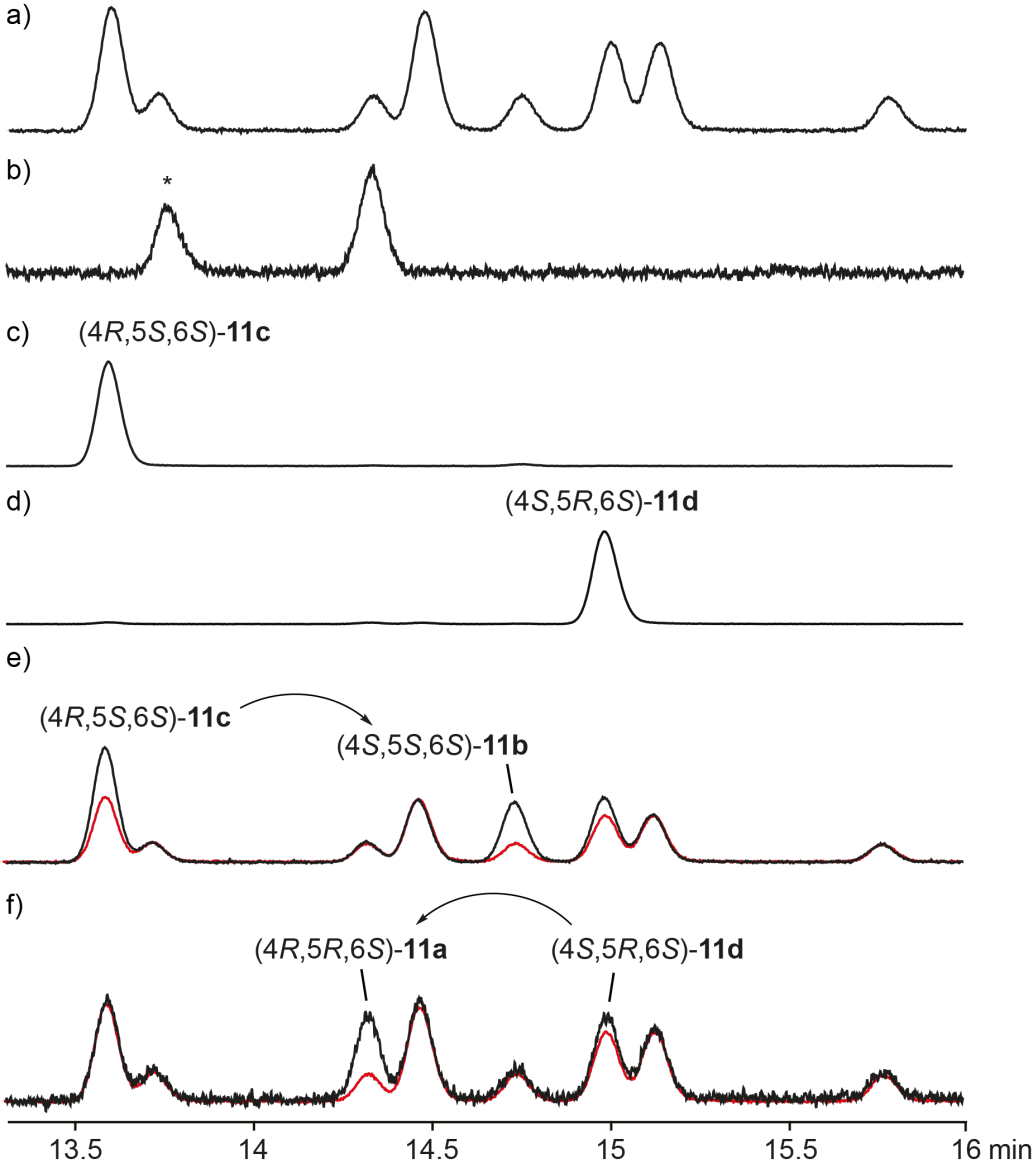
Gas chromatographic analysis of **11** on a homochiral stationary phase. a) Synthetic mixture of all eight stereoisomers (Scheme 4), b) headspace extract from *D. clavata*, c) enantioselectively synthesised (4*R*,5*S*,6*S*)-**11c** (Scheme 5), d) enantioselectively synthesised (4*S*,5*R*,6*S*)-**11d** (Scheme 5), e) coinjection of a) and epimerisation products of (4*R*,5*S*,6*S*)-**11c** (Scheme 6), and f) coinjection of a) and epimerisation products of (4*S*,5*R*,6*S*)-**11d** (Scheme 6). The peak labelled with asterisk represents the second main natural product in the headspace extracts from *D. clavata*, also labelled by asterisk in Figure 1. For comparison, the relative proportions of the eight stereoisomers obtained by synthesis according to Scheme 4 are indicated by the red curve in e) and f).

The mass spectrum of one of the two main compounds (**11a**) in the headspace extracts (Figure 2B) showed some fragment ions that were also observed for **10**, suggesting that the two volatiles may be structurally related. The fragment ions at *m*/*z* = 57 and *m*/*z* = 86 supported the structure of a 4-methyl-3-ketone (these ions would arise by α-cleavage and McLafferty rearrangement). Biosynthetically, **10** is a tetraketide, and if the dehydration step to install the C=C double bond in **10** would be omitted, this would lead to the hydroxy-ketone **11a** for which a higher retention time than for **10** would be expected. The structure of such an alcohol was further supported by the fragment ion at *m*/*z* = 115 that may result from an α-cleavage next to the alcohol function. To verify this structural proposal for **11a**, racemic 2-methylbutanal (**18**) was reacted in an aldol addition with the enolate anion of pentan-3-one (**23**) which produced a racemic mixture of all four diastereomers **11a**–**d** (Scheme 4). All eight stereoisomers of **11** were separable by GC on a homochiral stationary phase, one of which matched the natural product in terms of same retention times and mass spectra (Figure 3).

**Scheme 5:**
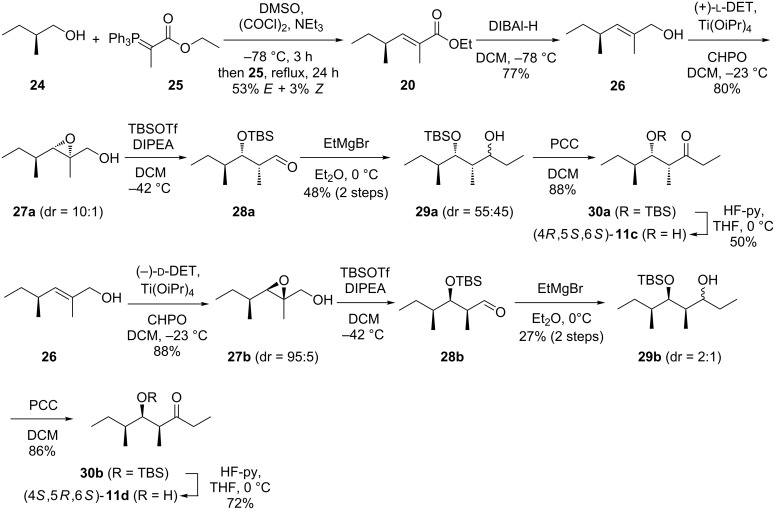
Enantioselective synthesis of (4*R*,5*S*,6*S*)-**11c** and (4*S*,5*R*,6*S*)-**11d**.

To clarify the relative and absolute configuration of the natural stereoisomer of **11** an enantioselective synthesis was performed (Scheme 5). The alcohol (*S*)-2-methylbutan-1-ol (**24**) was converted into the corresponding aldehyde by Swern oxidation, followed by the addition of **25** to yield the esters (2*E*,4*S*)- and (2*Z*,4*S*)-**20** as a mixture of diastereomers (ca. 15:1) that was readily separated by column chromatography. The *E* isomer was reduced with DIBAl-H to **26** that was converted into the epoxide **27a** by Sharpless epoxidation with (+)-L-DET. Treatment with TBSOTf and Hünig’s base resulted in opening of the epoxide with concomitant hydride migration to yield **28a**. The stereochemical course for this reaction has been reported by Jung and D’Amico [37] and proceeds with inversion of configuration at C-2. Grignard reaction with ethylmagnesium bromide to **29a**, PCC oxidation to **30a** and deprotection with HF-pyridine gave access to (4*R*,5*S*,6*S*)-**11c** with an overall yield of 7% via seven steps. The ^13^C NMR data in CDCl_3_ (cf. Supporting Information File 1) were identical to previously reported data [38].

**Scheme 6:**
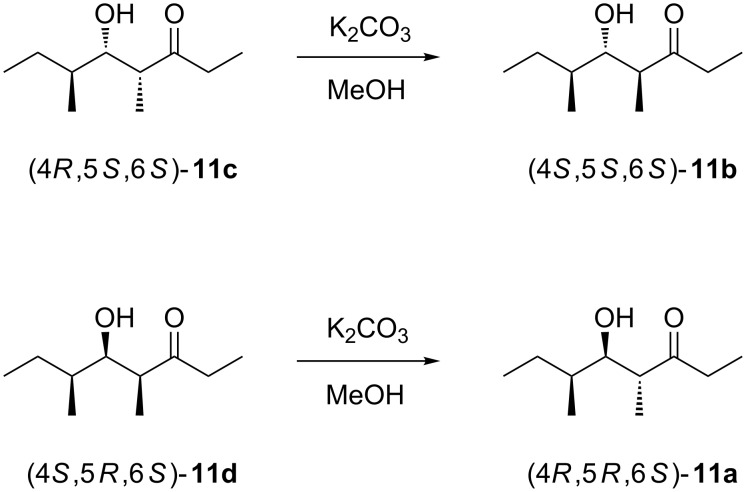
Epimerisations of (4*R*,5*S*,6*S*)-**11c** and (4*S*,5*R*,6*S*)-**11d** under basic conditions.

The allyl alcohol **26** was also used in a Sharpless epoxidation with (−)-D-DET to give **27b** that was converted into (4*S*,5*R*,6*S*)-**11d** via the same sequence of steps with a total yield of 6% via seven steps. Starting from the alcohol **24**, two more stereoisomers, (4*R*,5*R*,6*S*)-**11a** and (4*S*,5*S*,6*S*)-**11b**, would be accessible via the same route from (2*Z*,4*S*)-**20**, but this compound was not obtained in sufficient quantity for a practical approach towards **11a** and **11b**. Therefore, these two stereoisomers were obtained by epimerisation at the α-carbon (C-4) under mildly basic conditions (Scheme 6). Following this approach, the epimerisation of (4*R*,5*S*,6*S*)-**11c** yielded (4*S*,5*S*,6*S*)-**11b**, while epimerisation of (4*S*,5*R*,6*S*)-**11d** gave (4*R*,5*R*,6*S*)-**11a**. The epimerisation products were added to the synthetic mixture of all eight stereoisomers, showing by enantioselective GC analysis that the natural product was identical to (4*R*,5*R*,6*S*)-**11a** (Figure 3).

**Figure 4:**
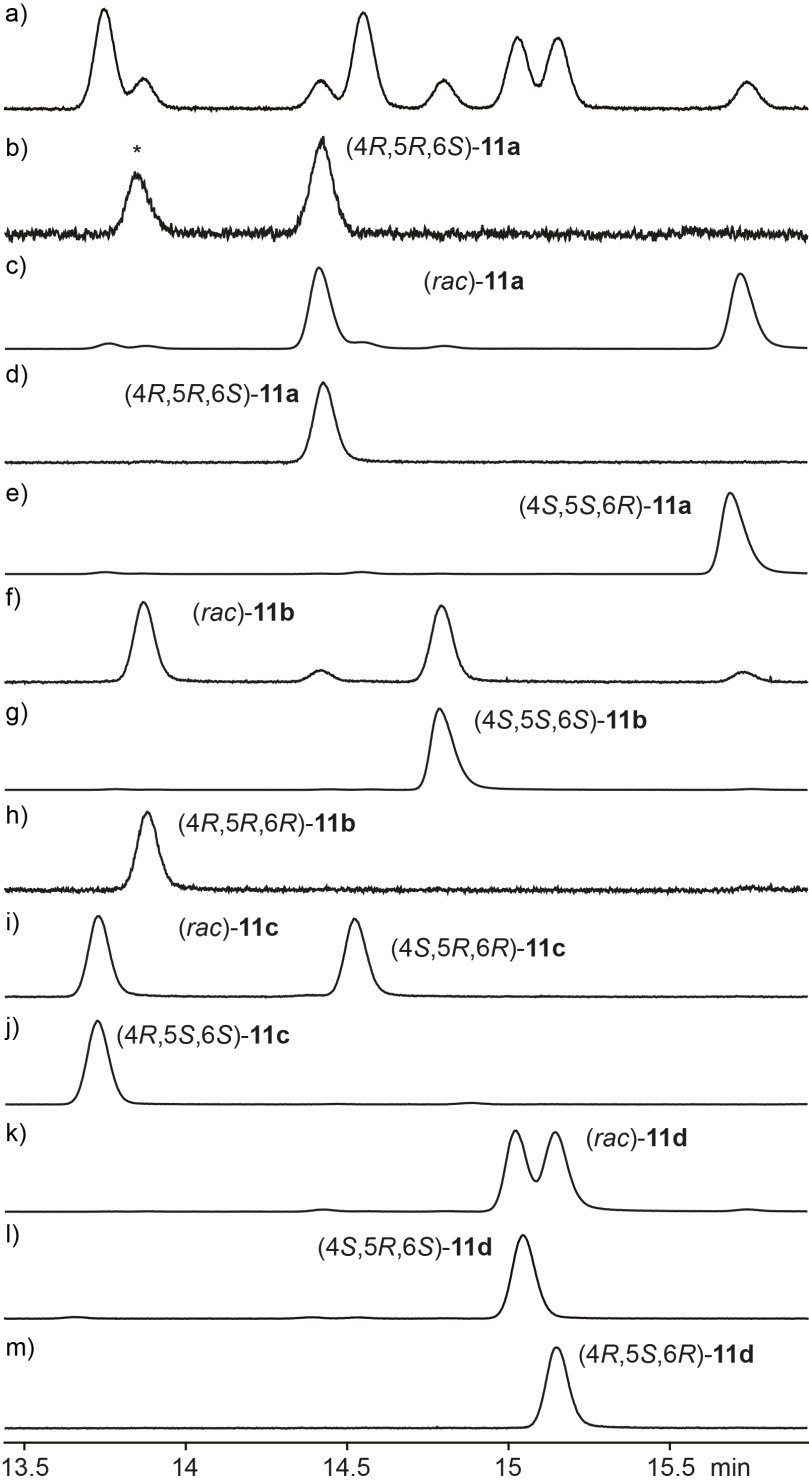
Gas chromatographic analysis of **11** on a homochiral stationary phase. a) Synthetic mixture of all eight stereoisomers (Scheme 4), b) headspace extract from *D. clavata*, and c) – m) pure racemates and enantiomers of **11a**–**d**. Compound (4*R*,5*S*,6*S*)-**11c** was obtained by enantioselective synthesis (Scheme 5), the pure racemates of **11a**–**d** and the pure enantiomers of **11a**, **11b** and **11d** were obtained by chromatographic separation from the synthetic mixture of all eight stereoisomers. The peak labelled with asterisk represents the second main natural product in the headspace extracts from *D. clavata*, also labelled by asterisk in Figure 1.

For the chemical characterisation of all eight stereoisomers of **11** the mixture obtained by the reaction shown in Scheme 4 was separated by extensive chromatographic purification. Compound **11c** was readily separated from the mixture of **11a**, **11b** and **11d** by simple column chromatography on silica gel. Preparative reversed-phase HPLC allowed for a separation of all three compounds **11a**, **11b** and **11d**. Subsequent preparative HPLC using a homochiral stationary phase gave access to the pure enantiomers of these three compounds, but unfortunately not of **11c**. However, as described above, enantiomerically pure (4*R*,5*S*,6*S*)-**11c** was obtained by enantioselective synthesis, and the peaks for the enantiomers of **11c** in the GC analysis on a homochiral stationary phase could be readily assigned by comparison to synthetic (4*R*,5*S*,6*S*)-**11c**. Finally, a structure could be assigned to each of the eight peaks observed in this analysis, again confirming the structure of (4*R*,5*R*,6*S*)-**11a** for the natural product from *D. clavata* (Figure 4). The ^13^C NMR data of all four stereoisomers **11a**–**d** are summarised in Table 2.

**Table 2:** ^13^C NMR data (chemical shifts in ppm) for **11a**–**d** (125 MHz, C_6_D_6_).

carbon	**11a**	**11b**	**11c**	**11d**

C-1	7.7	7.7	7.8	7.8
C-2	36.1	36.2	34.6	34.9
C-3	215.0	215.6	215.4	214.5
C-4	48.8	47.7	47.3	48.2
C-5	75.8	78.4	74.6	74.7
C-6	37.0	38.1	37.3	37.5
C-7	27.1	23.6	25.4	26.2
C-8	12.0	11.8	9.3	11.2
4-Me	12.4	14.7	11.3	11.4
6-Me	13.9	16.3	15.0	14.5

**Scheme 7:**
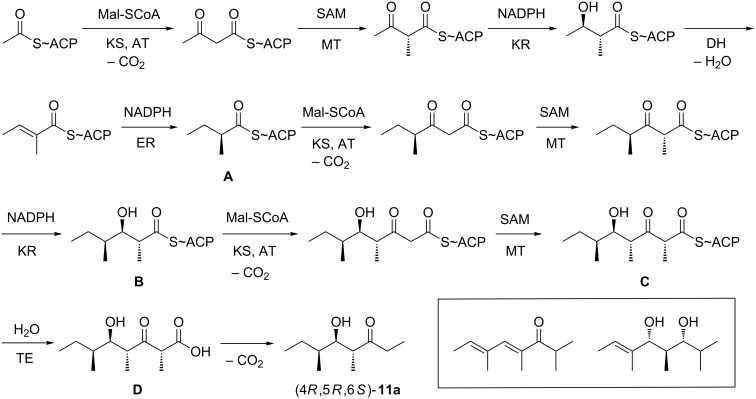
Proposed biosynthesis for (4*R*,5*R*,6*S*)-**11a**.

A proposed biosynthetic pathway to (4*R*,5*R*,6*S*)-**11a** is shown in Scheme 7 that is likely performed by a typical fungal iterative polyketide synthase (PKS). Starting from acyl-carrier-protein (ACP) bound acetate a first elongation step with malonyl-SCoA (Mal-SCoA) catalysed by an acyl transferase (AT) and a ketosynthase (KS) domain yields acetoacetyl-SACP. This may be followed by SAM-dependent C-methylation by a methyl transferase domain (MT). The stereochemical course for this reaction can be inferred from the 4*R*-configuration of the final product **11a**, if indeed an iterative PKS is involved that should have the same stereochemical course for the corresponding reactions in each chain extension step. A keto-reductase (KR) installs the 3-hydroxy group with the same stereochemistry as observed at C-5 in **11a**. This is followed by elimination of water by a dehydratase domain (DH) and reduction of the C=C double bond by an enoyl reductase (ER) with installation of the stereocentre in intermediate **A** corresponding to C-6 of **11a**. The next chain extension with malonyl-SCoA and methylation proceeds with the same stereochemical courses as discussed above, but stops after action of the KR to yield intermediate **B** in which all the stereocentres that occur in **11a** are already defined. A third extension with malonyl-SCoA and methylation gives rise to intermediate **C** that can be released, e.g., by a thioesterase to the β-keto acid **D**, followed by spontaneous decarboxylation to **11a**. Two structurally related molecules to **11a** have been reported from endophytic *Nodulisporium* spp. (shown in the box in Scheme 7) [28,39] that may be formed by a similar PKS. Further investigations are required to identify the PKSs for this family of metabolites and to confirm the hypothetical biosynthesis as shown in Scheme 7.

**Figure 5:**
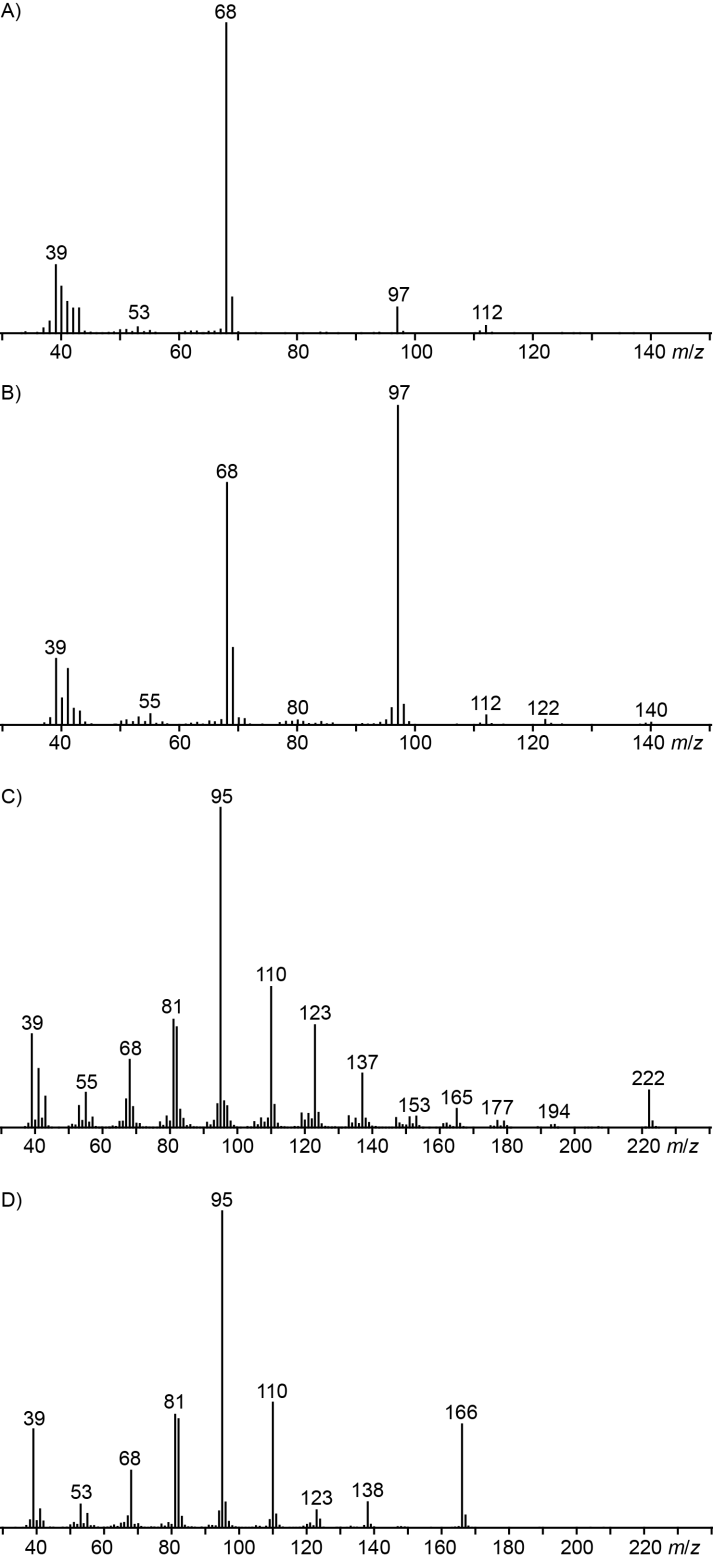
Mass spectra of a) 6-methyl-5,6-dihydro-2*H*-pyran-2-one (**9**), b) 6-propyl-5,6-dihydro-2*H*-pyran-2-one, c) 6-nonyl-2*H*-pyran-2-one (**17**), and d) 6-pentyl-2*H*-pyran-2-one.

**Scheme 8:**
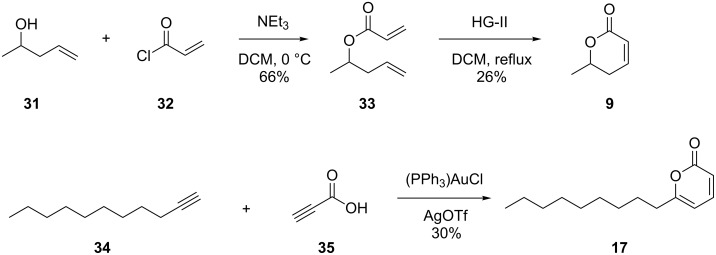
Synthesis of 6-methyl-5,6-dihydro-2*H*-pyran-2-one (**9**) and 6-nonyl-2*H*-pyran-2-one (**17**).

The volatile **9**, a compound emitted in small amounts by *D. clavata*, showed a mass spectrum that was not included in our database (Figure 5A). However, the mass spectrum of 6-propyl-5,6-dihydro-2*H*-pyran-2-one (Figure 5B) is very similar, and the mass difference for the molecular ion of 28 Da suggested the structure of 6-methyl-5,6-dihydro-2*H*-pyran-2-one for compound **9**. This compound was synthesised by esterification of pent-4-en-2-ol (**31**) with acryloyl chloride (**32**) to **33**, followed by ring-closing metathesis using the Hoveyda–Grubbs catalyst of the second generation (Scheme 8). The synthetic material proved to be identical to the volatile of *D. clavata*, thereby establishing its identity.

Finally, major amounts of a compound were emitted by *D. clavata* whose mass spectrum showed strong similarities to the mass spectrum of **2** (Figure 5C and 5D). The mass difference of 56 Da for the molecular ion pointed to four additional methylene units, suggesting the structure of 6-nonyl-2*H*-pyran-2-one (**17**). For comparison a synthetic reference compound was prepared from undec-1-yne (**34**) and propiolic acid (**35**) in a gold-catalysed reaction developed by Schreiber and co-workers [40] (Scheme 8). Synthetic **17** and the volatile from *D. clavata* showed the same mass spectrum and retention time, confirming their identity. The newly identified natural product **17** further extends the number of known 6-alkyl-2*H*-pyran-2-ones: While the mixture of 6-propyl-, 6-pentyl- and 6-heptyl-2*H*-pyran-2-one occurs in *Trichoderma viride* [41], higher homologs have not been reported as natural products so far.

### Biological characterisation of compounds

Three of the volatile metabolites identified from *D. clavata* (compounds **9**, **14** and **17**) were evaluated for antimicrobial and cytotoxic activities using a standard panel of fungi and bacteria, as well as the murine cell line L929 (Table 3). The 2-pyrone **17** exhibited a moderate antifungal and weak antibacterial effect, while the lactone **9** was moderately cytotoxic, but devoid of significant antimicrobial activity. The chlorinated aromatic compound **14** only weakly inhibited the sensitive test organisms *Chromobacterium violaceum* and *Mucor hiemalis*.

**Table 3:** In vitro antibacterial, antifungal and cytotoxic activity of compounds **9**, **14** and **17** in comparison with positive controls.

Test organism	**9**^a^	**14**^a^	**17**^a^	Reference

Fungi				

*Candida albicans* DSM 1665	–	–	16.6	33.3^b^
*Mucor hiemalis* DSM 2656	33.3	–	16.6	5.25–16.6^b^
*Pichia anomala* DSM 70255	–	–	33.3	33.3^b^
*Rhodotorula glutinis* DSM 10134	–	–	16.6	0.52–16.7^b^
*Schizosaccharomyces pombe* DSM 70572	–	–	16.6	42.0–67.0^b^

Bacteria				

*Bacillus subtilis* DSM 10	–	–	67.0	6.7–8.3^c^
*Chromobacterium violaceum* DSM 30191	67.0	–	–	0.83–8.3^c^
*Escherichia coli* DSM 1116	–	–	–	0.83^c^
*Micrococcus luteus* DSM 20030	–	–	–	0.42^c^
*Mycobacterium* sp. DSM 43270	–	–	–	2.1^c^
*Pseudomonas aeruginosa* DSM 50071	–	–	–	16.6–21.0^d^
*Staphylococcus aureus* DSM 346	–	–	67.0	0.1–0.21^c^
Eukaryotic cell line				
Murine cell line L929	>10	6	>10	1 nm^e^

^a^For compound testings against bacteria and fungi minimum inhibitory concentrations (MIC) in μg mL^−1^ are given, for cytotoxicity testings against the murine cell line the IC_50_ is given in μg mL^−1^. Reference compounds: ^b^nystatin, ^c^oxytetracyclin hydrochloride, ^d^gentamycin, ^e^epothilon B.

## Conclusion

The current study provides evidence that manifold new volatiles may be encountered in the future through a systematic study of Xylariales and other predominant fungal endophytes. As many of these fungi show interesting activities in screening approaches for biocontrol agents, and some of them are even under development for commercial applications, such studies not only complement the knowledge on the metabolic capabilities, but may even become mandatory in order to provide final proof for the safety of the organisms. There have been some studies on VOC-producing xylarialean endophytes with significant activities against competing microbes, but the volatile profiles of these biocontrol candidates were only evaluated using databases like NIST, which can only serve to detect and identify known compounds. The current study demonstrates the need of chemical synthesis for rigorous identification of new compounds. Some of these metabolites were tested for biological effects and found to display only weak activities in biological systems, providing evidence for their safety. In addition, none of the volatiles detected here represents a metabolite that is biosynthetically linked to a known class of hazardous mycotoxins. Similar studies on other fungal cultures with proven initial antagonistic activities that can be related to VOCs will probably be rewarding.

## Supporting Information

File 1 Experimental details, synthetic procedures, and spectroscopic data for synthetic compounds.
