# Supplementary material for: Volatiles from the tropical ascomycete Daldinia clavata (Hypoxylaceae, Xylariales)
Source: Beilstein J Org Chem. 2018 Jan 12;14:135–47. doi: 10.3762/bjoc.14.9 (PMC5789425; doi:10.3762/bjoc.14.9)
Supplement: File 1 — Experimental details, synthetic procedures, and spectroscopic data for synthetic compounds. [file Beilstein_J_Org_Chem-14-135-s001.pdf]

Supporting Information

for

Volatiles from the tropical ascomycete *Daldinia clavata* (Hypoxylaceae, Xylariales)

Tao Wang[1], Kathrin I. Mohr[2], Marc Stadler[2] and Jeroen S. Dickschat*[1]

Address: [1]Kekulé-Institut für Organische Chemie, Universität Bonn, Gerhard-Domagk-Straße 1, 53121 Bonn, Germany and [2]Abteilung für Mikrobielle Wirkstoffe, Helmholtz-Zentrum für Infektionsforschung, Inhoffenstraße 7, 38124 Braunschweig, Germany

Email: Jeroen S. Dickschat - dickschat@uni-bonn.de

* Corresponding author

Experimental details, synthetic procedures, and spectroscopic data for synthetic compounds

Strain and culture conditions

The culture of *Daldinia clavata* Henn. used in the current study was isolated from ascospores of a specimen collected by Cony Decock in Gabon, Ogooué-Ivindo Province, Parc National de l'Ivindo, Réserve

Intégrale d'Ipassa, Ipassa Biological Station, on unidentified angiosperm wood and its characteristics have been reported in the last world monography of the genus *Daldinia* [1]. The strain and the corresponding specimen are deposited in the herbarium and culture collection of BCCM/MUCL, Louvain-la-Neuve, Belgium under designation no. MUCL 47435. The culture is also maintained at the Helmholtz Centre for Infection Research in the personal collection of M.S. (designation no. STMA 06094). The strain was grown on YMG medium as previously described [2].

CLSA sampling

The volatiles emitted by agar plate cultures of *Daldinia clavata* were trapped on charcoal filters through the closed-loop stripping apparatus (CLSA) headspace technique. After 16 to 24 hours of collection at room temperature (20 °C) and under natural day and night light conditions, the charcoal filters were extracted with dichloromethane (50 μL) and the extracts were directly analysed by GC–MS.

General synthetic methods

Chemicals were purchased Sigma Aldrich Chemie GmbH (Steinheim, Germany) and Acros Organics (Geel, Belgium) and used without further purification. Solvents were distilled prior to usage and dried according to

standard methods. Moisture or oxygen sensitive reactions were carried out in flame-dried flasks under nitrogen or argon atmosphere. Thin-layer chromatography was performed using Polygram Sil G/UV_{254} plates from Macherey-Nagel (Düren, Germany). Compounds were visualised under UV-light (λ = 254 nm) or by chemical staining with molybdophosphoric acid (10 g molybdophosphoric acid in 100 mL ethanol). Column chromatography was performed using silica gel 60 (0.04–0.063 mm, 230–400 mesh) purchased from Macherey-Nagel.

Spectroscopy

NMR spectra were recorded on Bruker AV I (400 MHz), AV III HD Prodigy (500 MHz) and AV III HD Cryo (700 MHz) spectrometers, and were referenced against $CDCl_3$ (δ = 7.26 ppm) and C_6D_6 (δ = 7.16 ppm) for ^{1}H NMR, and $CDCl_3$ (δ = 77.16 ppm) and C_6D_6 (δ = 128.06 ppm) for ^{13}C NMR. IR Spectra were measured with a Bruker Alpha FT-IR spectrometer. The intensities of the peaks were described as s (strong), m (medium), and w (weak). UV–vis spectra were recorded on a Cary 100 UV–vis spectrometer (Agilent). Optical rotations were measured on a P8000 polarimeter (Krüss).

GC–MS

GC–MS analyses were carried out on an Agilent 7890A connected with an Agilent 5975C inert mass detector (Hewlett-Packard Company, Wilmington, USA) fitted with a non-polar BPX-5 (25 m, 0.22 mm i. d., 0.25 μm film, SGE Inc., Melbourne, Australia) or HP5-MS fused silica capillary column (30 m, 0.25 mm i. d., 0.25 μm film, Agilent). Conditions were inlet pressure: 77.1 kPa, He 23.3 mL min^{-1}; injection volume: 1.5 μL; injector operation mode: splitless (60 s valve time); carrier gas: He at 1.2 mL min^{-1}; GC program: 5 min at 50 °C, then increasing with 5 °C min^{-1} to 320 °C; transfer line 300 °C; electron energy 70 eV. Retention indices (*I*) were determined from a homologous series of *n*-alkanes (C_8–C_{38}).

Gas chromatography using a chiral stationary phase was performed with a CP-ChiraSil-Dex CB capillary column (25 m, 0.25 mm i. d., 0.36 μm film, Agilent) with the following temperature program: 100 °C, then increasing with 1 °C min^{-1} to 150 °C, then with 20 °C min^{-1} to 245 °C.

High-performance liquid chromatography (HPLC)

High-performance liquid chromatography was performed using a Knauer GmbH (Berlin, Germany) system: two pumps P-1 HPLC plus (max. 750 bar), oven T-1 with two integrated 6-port valves, photodiode array detector PDA-1 (190–1000 nm); column: KNAUER Eurospher II 100-5

C18 (3 μm, 16.0 mm × 250 mm); solvent: acetonitrile/water (35/65); flow rate: 16.0 mL min^{-1}; pressure: 242 bar, temperature: 25 °C. For separation of enantiomers the same system was used, equipped with a DAICEL Chiralpak IA column (5 μm, 4.6 mm × 250 mm); solvent: *n*-hexane/2-propanol (98/2); flow rate: 1.0 mL min^{-1}; pressure: 34 bar, temperature: 25 °C.

Ethyl (*E*)- and (*Z*)-2,4-dimethylhex-2-enoate (20)

To a solution of LiCl (1.02 g, 24 mmol), DBU (3.04 g, 20 mmol) and ethyl 2-(diethoxyphosphoryl)propanoate (5.72 g, 24 mmol) in acetonitrile (240 mL), 2-methylbutanal (1.72 g, 20 mmol) was added and stirred at room temperature for one hour. The reaction was quenched with distilled water and extracted three times with diethyl ether. The collected organic phases were dried with $MgSO_4$ and concentrated in vacuo. GC analysis of the crude product showed the presence of two stereoisomers in a ca. 10:1 ratio. Purification by flash column chromatography on silica gel afforded ethyl (*E*)-2,4-dimethylhex-2-enoate ((*E*)-**20**, 1.30 g, 7.67 mmol, 38%) and ethyl (*Z*)-2,4-dimethylhex-2-enoate ((*Z*)-**20**, 15 mg, 0.088 mmol, 1%) as colourless oils.

Analytical data for (*E*)-**20**: TLC (hexane/ethyl acetate = 20:1): R_f = 0.31. GC (BPX-5): I = 1152. ^{1}H NMR (400 MHz, $CDCl_3$, TMS): δ = 6.52 (dq, $^4J_{H,H}$ = 1.4 Hz, $^3J_{H,H}$ = 10.0 Hz, 1H, CH), 4.18 (q, $^3J_{H,H}$ = 7.1 Hz, 2H, CH_2),

2.45-2.34 (m, 1H, CH), 1.83 (d, $^4J_{H,H}$ = 1.4 Hz, 3H, CH_3), 1.46-1.25 (m, 2H, CH_2), 1.29 (t, $^3J_{H,H}$ = 7.3 Hz, 3H, CH_3), 0.99 (d, $^3J_{H,H}$ = 6.8 Hz, 3H, CH_3), 0.85 (t, $^3J_{H,H}$ = 7.4 Hz, 3H, CH_3) ppm. ^{13}C NMR (100 MHz, $CDCl_3$, TMS): δ = 168.6 (C_q), 147.8 (CH), 126.6 (C_q), 60.4 (CH_2), 34.9 (CH), 29.7 (CH_2), 19.7 (CH_3), 14.3 (CH_3), 12.5 (CH_3), 11.9 (CH_3) ppm. IR (ATR): $\tilde{\nu}$ = 2962 (m), 2930 (m), 2874 (m), 1709 (w), 1650 (m), 1458 (m), 1367 (m), 1316 (m), 1269 (w), 1235 (w), 1152 (w), 1033 (m), 995 (m), 924 (m), 871 (m), 776 (s), 748 (w), 532 (s) cm^{-1}. UV-Vis (CH_2Cl_2): λ_{max} (log ε) = 231 (4.71) nm. MS (EI, 70 eV): *m*/*z* (%) = 170 (60) $[M]^+$, 125 (70), 123 (21), 113 (87), 109 (57), 102 (28), 97 (41), 96 (70), 95 (89), 87 (30), 85 (28), 81 (43), 73 (20), 69 (44), 67 (89), 56 (33), 55 (100), 53 (44), 43 (72), 41 (97), 39 (55).

Analytical data for (*Z*)-**20**: TLC (hexane/ethyl acetate = 20:1): R_f = 0.29. GC (BPX-5): *I* = 1081. ^{1}H NMR (400 MHz, $CDCl_3$, TMS): δ = 5.63 (dq, $^4J_{H,H}$ = 1.5 Hz, $^3J_{H,H}$ = 10.1, 1H, CH), 4.18 (q, $^3J_{H,H}$ = 7.1 Hz, 2H, CH_2), 3.04-2.93 (m, 1H, CH), 1.88 (d, $^4J_{H,H}$ = 1.5 Hz, 3H, CH_3), 1.38-1.21 (m, 2H, CH_2), 1.29 (t, $^3J_{H,H}$ = 7.1 Hz, 3H, CH_3), 0.96 (d, $^3J_{H,H}$ = 6.6 Hz, 3H, CH_3), 0.84 (t, $^3J_{H,H}$ = 7.5 Hz, 3H, CH_3) ppm. ^{13}C NMR (100 MHz, $CDCl_3$): δ = 168.5 (C_q), 148.3 (CH), 126.1 (C), 60.0 (CH_2), 35.0 (CH), 30.1 (CH_2), 20.8 (CH_3), 20.2 (CH_3), 14.2 (CH_3), 11.8 (CH_3) ppm. IR (ATR): $\tilde{\nu}$ = 2962 (m), 2930 (m), 2873 (m), 1715 (w), 1646 (m), 1456 (m), 1372 (m), 1326 (s), 1262 (m), 1221 (w), 1171 (w), 1096 (w), 1044 (m), 1023 (m), 971

(m), 889 (s), 771 (m), 594 (s) cm^{-1}. UV-Vis (CH_2Cl_2): λ_{max} (log ε) = 229 (4.71) nm. MS (EI, 70 eV): *m*/*z* (%) = 170 (56) $[M]^+$, 125 (72), 123 (21), 113 (69), 109 (61), 102 (25), 97 (34), 96 (59), 87 (25), 81 (40), 69 (39), 67 (81), 56 (32), 55 (79), 53 (41), 43 (71), 41 (87), 39 (50).

(*E*)-2,4-Dimethylhex-2-en-1-ol (21)

To a solution of (*E*)-**20** (1.16 g, 6.8 mmol) in dry dichloromethane (27.2 mL), DIBAl-H (13.6 mL, 13.6 mmol, 1 M in hexane) was added dropwise at −78 °C and stirred at −78 °C overnight. Then, saturated aqueous NH_4Cl solution was added and the mixture was allowed to warm to room temperature. The reaction mixture was filtered through Celite and extracted three times with dichloromethane. The combined extracts were dried with $MgSO_4$ and concentrated in vacuo. The crude product was purified by flash column chromatography on silica gel to yield (*E*)-2,4-dimethylhex-2-en-1-ol (**21**, 481 mg, 3.75 mmol, 55%) as a colourless oil. TLC (hexane/ethyl acetate = 5:1): R_f = 0.32. GC (HP-5MS): *I* = 996. ^1H NMR (400 MHz, $CDCl_3$, TMS): δ = 5.16 (dtq, $^4J_{H,H}$ = 1.3 Hz, $^4J_{H,H}$ = 1.3 Hz, $^3J_{H,H}$ = 9.5 Hz, 1H, CH), 3.99 (d, $^4J_{H,H}$ = 1.2 Hz, 2H, CH_2), 2.33-2.22 (m, 1H, CH), 1.66 (d, $^4J_{H,H}$ = 1.4 Hz, 3H, CH_3), 1.45 (s, 1H, OH), 1.39-1.17 (m, 2H, CH_2), 0.93 (d, $^3J_{H,H}$ = 6.7 Hz, 3H, CH_3), 0.83 (t, $^3J_{H,H}$ = 7.4 Hz, 3H, CH_3) ppm. ^{13}C NMR (100 MHz, $CDCl_3$, TMS): δ = 133.4 (CH), 132.7 (C_q), 69.0 (CH_2), 33.7 (CH), 30.2 (CH_2), 20.6 (CH_3), 13.8 (CH_3),

11.9 (CH_3) ppm. IR (ATR): $\tilde{\nu}$ = 3321 (m), 2960 (m), 2922 (m), 2872 (m), 1454 (m), 1377 (m), 1260 (m), 1237 (w), 1091 (w), 1003 (s), 949 (m), 863 (m), 808 (m), 771 (w), 602 (w) cm^{-1}. UV-Vis (CH_2Cl_2): λ_{max} (log ε) = 231 (3.24) nm. MS (EI, 70 eV): *m*/*z* (%) = 128 (7) $[M]^+$, 97 (53), 95 (23), 83 (7), 81 (13), 79 (7), 73 (77), 71 (32), 69 (18), 67 (13), 58 (10), 57 (17), 56 (10), 55 (52), 44 (10), 43 (100), 41 (25), 40 (13), 39 (11).

(*E*)-4,6-Dimethyloct-4-en-3-ol (22)

To a solution of **21** (481 mg, 3.75 mmol) in anhydrous dichloromethane (7.5 mL), pyridium chlorochromate (968 mg, 4.5 mmol) was added at room temperature and the mixture was stirred overnight. The reaction mixture was filtered through silica gel, concentrated in vacuo and used for the next step without purification.

To a solution of the obtained aldehyde in anhydrous diethyl ether (7.5 mL), freshly prepared EtMgBr (7.5 mL, 7.50 mmol, 1 M in Et_2O) was added dropwise at 0 °C and the reaction was stirred at 0 °C for two hours. Then, distilled water was added slowly. The mixture was extracted three times with Et_2O, the combined extracts were dried over $MgSO_4$ and concentrated in vacuo. Purification by flash column chromatography on silica gel gave a mixture of two diastereomers of (*E*)-4,6-dimethyloct-4-en-3-ol (**22**, 328 mg, 2.10 mmol, 56%, *dr* 1:1) as a colourless oil.

Analytical data for mixture of diastereomers: TLC (hexane/ethyl acetate = 10:1): R_f = 0.38. ^{1}H NMR (500 MHz, $CDCl_3$, TMS): δ = 5.15-5.09 (m, 1H, CH), 3.90 (t, $^3J_{H,H}$ = 6.8 Hz, 1H, CH), 2.34-2.23 (m, 1H, CH), 1.59 (br s, 3H, CH_3), 1.57-1.47 (m, 2H, CH_2), 1.39-1.14 (m, 2H, CH_2), 0.95-0.90 (m, 3H, CH_3), 0.86-0.79 (m, 6H, 2xCH_3) ppm. ^{13}C NMR (125 MHz, $CDCl_3$, TMS): δ = 135.5, 135.4 (C_q), 133.8, 133.2 (CH), 79.8, 79.4 (CH), 33.8, 33.7 (CH), 30.4, 30.3 (CH_2), 27.7, 27.6 (CH_2), 20.8, 20.7 (CH_3), 12.0 (CH_3), 11.5, 11.0 (CH_3), 10.1, 10.0 (CH_3) ppm. IR (ATR): $\tilde{\nu}$ = 3359 (m), 2960 (s), 2930 (m), 2873 (m), 1457 (m), 1376 (m), 1315 (w), 1261 (w), 1237 (w), 1093 (m), 1050 (m), 991 (s), 964 (m), 890 (m), 866 (m), 830 (w), 776 (m), 620 (w), 546 (m) cm^{-1}. UV-Vis (CH_2Cl_2): λ_{max} (log ε) 230 (3.19) nm.

Diastereomer A: GC (HP-5MS): *I* = 1111. MS (EI, 70 eV): *m*/*z* (%) = 156 (3) $[M]^+$, 128 (43), 111 (10), 110 (95), 100 (100), 98 (14), 96 (13), 92 (8), 86 (29), 84 (32), 82 (17), 78 (13), 77 (11), 73 (14), 72 (82), 70 (45), 68 (28), 58 (91), 56 (41), 44 (90), 42 (29), 41 (30).

Diastereomer B: GC (HP-5MS): *I* = 1115. MS (EI, 70 eV): *m*/*z* (%) = 156 (2) $[M]^+$, 128 (45), 110 (11), 109 (96), 99 (100), 97 (12), 85 (20), 83 (34), 81 (20), 73 (11), 71 (76), 69 (40), 67 (27), 57 (80), 55 (35), 44 (34), 43 (47), 41 (28), 40 (26).

(*E*)-4,6-Dimethyloct-4-en-3-one (10)

To a solution of alcohols **22** (312 mg, 2.00 mmol) in anhydrous dichloromethane (4.00 mL), pyridinium chlorochromate (520 mg, 2.40 mmol) was added and the reaction was stirred overnight at room temperature. The reaction mixture was filtered over silica gel, concentrated in vacuo and the crude product was purified by flash column chromatography on silica gel to afford (*E*)-4,6-dimethyl-4-octen-3-one (**10**, 114 mg, 0.74 mmol, 37%) as colourless oil.

TLC (hexane/ethyl acetate = 10:1): R_f = 0.53. GC (HP-5MS): *I* = 1140. ^{1}H NMR (400 MHz, $CDCl_3$, TMS): δ = 6.36 (dq, $^3J_{H,H}$ = 9.7 Hz, $^4J_{H,H}$ = 1.4 Hz, 1H, CH), 2.67 (q, $^3J_{H,H}$ = 7.3 Hz, 2H, CH_2), 2.52-2.41 (m, 1H, CH), 1.77 (d, $^4J_{H,H}$ = 1.3 Hz, 3H, CH_3), 1.49-1.27 (m, 2H, CH_2), 1.08 (t, $^3J_{H,H}$ = 7.3 Hz, 3H, CH_3), 1.01 (d, $^3J_{H,H}$ = 6.6 Hz, 3H, CH_3), 0.86 (t, $^3J_{H,H}$ = 7.4 Hz, 3H, CH_3) ppm. ^{13}C NMR (100 MHz, $CDCl_3$, TMS): δ = 202.9 (C_q), 147.7 (CH), 135.6 (C_q), 35.1 (CH), 30.4 (CH_2), 29.7 (CH_2), 19.7 (CH_3), 11.9 (CH_3), 11.6 (CH_3), 8.9 (CH_3) ppm. IR (ATR): $\tilde{\nu}$ = 2962 (m), 2931 (m), 2875 (m), 1670 (s), 1458 (m), 1375 (m), 1341 (m), 1262 (m), 1227 (m), 1132 (m), 1085 (m), 1051 (m), 1015 (m), 991 (m), 896 (m), 800 (m), 581 (w) cm^{-1}. UV-Vis (CH_2Cl_2): λ_{max} (log ε) = 308 (2.79), 233 (5.05) nm. MS (EI, 70 eV): *m*/*z* (%) = 154 (33) $[M]^+$, 155 (4), 126 (9), 125 (100), 106 (8), 97 (11), 96 (4), 84 (6), 81 (4), 69 (17), 67 (5), 57 (11), 56 (3), 55 (43), 53 (3), 43 (13), 41 (9), 40 (5).

Preparation of stereoisomers of 11

To a solution of diisopropylamine (2.02 g, 20.0 mmol) in anhydrous THF (50 mL) was added *n*-butyllithium (12.5 mL, 1.6 M in hexane, 20.0 mmol) at 0 °C. The mixture was stirred at 0 °C for 1 hour and subsequently cooled to −78 °C. Pentan-3-one (1.72 g, 20.0 mmol) in anhydrous THF (10 mL) was added slowly at −78 °C and the mixture was stirred at this temperature for another hour, followed by the addition of 2-methylbutanal (1.72 g, 20.0 mmol) in anhydrous THF (10 mL). Stirring at −78 °C was continued for one more hour. The mixture was warmed to room temperature, hydrolysed with saturated ammonium chloride solution and extracted three times with diethyl ether. The collected organic phases were dried over $MgSO_4$ and concentrated under reduced pressure to yield 5-hydroxy-4,6-dimethyloctan-3-one (2.34 g, 13.6 mmol, 68%) as a mixture of eight stereoisomers. Spectroscopic data are given below for the enantioselectively synthesised or chromatographically purified stereoisomers.

Ethyl (2*E*,4*S*)- and (2*Z*,4*S*)-2,4-dimethylhex-2-enoate (20)

To a solution of oxalyl chloride (3.81 g, 30.0 mmol) in anhydrous dichloromethane (16.6 mL), dimethyl sulfoxide (4.88 g, 62.5 mmol) was added dropwise at −78 °C. After stirring for 5 min, (*S*)-(−)-2-methylbutanol (2.20 g, 25.0 mmol) in anhydrous dichloromethane

(6.9 mL) was added to the reaction mixture. Stirring was continued for another 30 minutes at −78 °C. Then, triethylamine (12.7 g, 150 mmol) was added to the solution that was subsequently allowed to warm to room temperature. (Ethoxycarbonylethylidiene)triphenylphosphorane (10.9 g, 30 mmol) was added and the mixture was stirred under reflux for 24 h. The reaction was quenched by the addition of distilled water and extracted three times with dichloromethane. The combined extracts were dried with $MgSO_4$ and concentrated under reduced pressure. The crude product was purified by flash column chromatography on silica gel to afford ethyl (2*E*,4*S*)-2,4-dimethylhex-2-enoate ((2*E*,4*S*)-**20**, 2.27 g, 13.34 mmol, 53%) and small amounts of ethyl (2*Z*,4*S*)-2,4-dimethylhex-2-enoate ((2*Z*,4*S*)-**20**, 0.13 g, 0.76 mmol, 3%) as colourless oils.

Analytical data for (2*E*,4*S*)-**20** were identical to those reported above for racemic (*E*)-**20**. Optical rotation: $[\alpha]_D^{20}$ = +28.4 (*c* 0.1, ethanol).

Analytical data for (2*E*,4*S*)-**20** were identical to those reported above for racemic (*Z*)-**20**. Optical rotation: $[\alpha]_D^{20}$ = +11.8 (*c* 0.1, ethanol).

(2*E*,4*S*)-2,4-Dimethylhex-2-en-1-ol (26)

To a solution of (2*E*,4*S*)-**20** (6.91 g, 40.7 mmol) in anhydrous dichloromethane (400 mL), DIBAl-H (1.2 M in toluene, 165.5 mL, 199 mmol) was added dropwise at −78 °C and the reaction was stirred at this temperature overnight. A saturated solution of NH_4Cl was added and the

mixture was warmed to room temperature. The colourless precipitate was filtered off and the reaction mixture was extracted three times with dichloromethane. The collected organic layers were dried over $MgSO_4$ and concentrated in vacuo. The residue was purified by flash column chromatography on silica gel to give (2*E*,4*S*)-2,4-dimethylhex-2-en-1-ol (**26**, 4.04 g, 31.5 mmol, 77%) as a colourless oil.

TLC (hexane/ethyl acetate = 5:1): R_f = 0.31. GC (BPX-5): *I* = 1068. ^{1}H NMR (400 MHz, $CDCl_3$, TMS): δ = 5.03 (br d, $^{3}J_{H,H}$ = 9.9 Hz, 1H, CH), 4.13 (dd, $^{2}J_{H,H}$ = 11.7 Hz, $^{4}J_{H,H}$ = 0.8 Hz, 1H, CH_2), 4.08 (dd, $^{2}J_{H,H}$ = 11.7 Hz, $^{4}J_{H,H}$ = 0.8 Hz, 1H, CH_2), 2.36-2.24 (m, 1H, CH), 1.78 (d, $^{4}J_{H,H}$ = 1.5 Hz, 3H, CH_3), 1.49 (br s, 1H, OH), 1.37-1.26 (m, 1H, CH_2), 1.23-1.11 (m, 1H, CH_2), 0.91 (d, $^{3}J_{H,H}$ = 6.6 Hz, 3H, CH_3), 0.81 (t, $^{3}J_{H,H}$ = 7.4 Hz, 3H, CH_3) ppm. ^{13}C NMR (100 MHz, $CDCl_3$): δ = 135.1 (C_q), 133.0 (CH), 61.9 (CH_2), 33.8 (CH), 30.4 (CH_2), 21.4 (CH_3), 21.3 (CH_3), 12.0 (CH_3) ppm. IR (ATR): $\tilde{\nu}$ = 3319 (w), 2960 (m), 2922 (m) 2872 (m), 1454 (m), 1376 (w), 1236 (w), 1115 (w), 1003 (s), 949 (w), 863 (w), 811 (w), 711 (w), 601 (w) cm^{-1}. UV-Vis (CH_2Cl_2): λ_{max} (log ε) = 227 (2.81), 221 (2.31) nm. MS (EI, 70 eV): *m*/*z* (%) = 110 (1) $[M-H_2O]^+$, 87 (7), 71 (6), 70 (6), 69 (22), 67 (7), 59 (7), 58 (29), 57 (67), 56 (10), 55 (51), 53 (15), 51 (10), 45 (39), 43 (74), 42 (25), 41 (67), 40 (12), 39 (100), 38 (7). Optical rotation: $[\alpha]_D^{20}$ = +10.4 (*c* 0.1, ethanol).

(2*S*,3*S*,4*S*)-2,3-Epoxy-2,4-dimethylhexan-1-ol (27a)

Molecular sieves (2.34 g, 4 Å) and dry dichloromethane (117 mL) were placed in a round-bottom flask and cooled to −10 °C. Then, (+)-diethyl L-tartrate (579 mg, 2.81 mmol), titanium tetraisopropoxide (665 mg, 2.34 mmol) and cumene hydroperoxide (4.45 g, 80% in cumene, 23.4 mmol) were added and the mixture was stirred for 10 minutes at −10 °C. The reaction mixture was subsequently cooled to −23 °C, followed by dropwise addition of the alcohol **26** (3.00 g, 23.4 mmol) in anhydrous dichloromethane (11.7 mL). Stirring was continued at −23 °C overnight. Distilled water was added and the reaction was stirred for another 30 minutes. The mixture was filtered through a pad of Celite, dried with $MgSO_4$, and concentrated in vacuo. The residue was purified by flash column chromatography on silica gel to yield the title compound **27a** as colourless oil (2.71 g, 18.8 mmol, 80%, *dr* > 10:1 by GC/MS analysis).

TLC (hexane/ethyl acetate = 1:1): R_f = 0.47. GC (BPX-5): *I* = 1106. ^{1}H NMR (400 MHz, $CDCl_3$, TMS): δ = 3.66 (dd, $^3J_{H,H}$ = 4.7 Hz, $^2J_{H,H}$ = 12.2 Hz, 1H, CH_2), 3.54 (dd, $^3J_{H,H}$ = 8.2, $^2J_{H,H}$ = 12.2 Hz, 1H, CH_2), 2.75 (d, $^3J_{H,H}$ = 8.9 Hz, 1H, CH), 2.06-1.99 (m, 1H, OH), 1.67-1.55 (m, 1H, CH), 1.41-1.27 (m, 2H, CH_2), 1.28 (s, 3H, CH_3), 0.96 (t, $^3J_{H,H}$ = 7.3 Hz, 3H, CH_3), 0.90 (d, $^3J_{H,H}$ = 6.6 Hz, 3H, CH_3) ppm. ^{13}C NMR (100 MHz, $CDCl_3$, TMS): δ = 65.6 (CH_2), 64.9 (CH), 60.3 (C_q), 34.1 (CH), 27.9 (CH_2), 15.6 (CH_3), 14.2 (CH_3), 11.3 (CH_3) ppm. IR (ATR): $\tilde{\nu}$ = 2960 (m), 2932 (m),

2881 (w), 2859 (w), 1708 (m), 1462 (w), 1383 (w), 1254 (w), 1182 (w), 1057 (m), 1022 (m), 962 (w), 940 (m), 835 (s), 810 (m), 773 (s), 674 (m) cm^{-1}. UV-Vis (CH_2Cl_2): λ_{max} (log ε) = 231 (2.04) nm. MS (EI, 70 eV): *m*/*z* (%) = 126 (0.5) $[M-H_2O]^+$, 111 (1), 97 (4), 87 (11), 75 (13), 74 (9), 71 (10), 70 (16), 69 (33), 67 (5), 59 (22), 58 (58), 57 (58), 56 (7), 55 (74), 53 (10), 51 (6), 45 (46), 43 (100), 42 (20), 41 (68), 40 (8), 39 (52), 38 (5). Optical rotation: $[\alpha]_D^{20}$ = +12.9 (*c* 0.1, ethanol).

(2*R*,3*R*,4*S*)-2,3-Epoxy-2,4-dimethylhexan-1-ol (27b)

This compound was prepared via the same procedure as described above for **27a**, only (−)-diethyl D-tartrate was used instead of (+)-diethyl L-tartrate. Starting from **26** (2.00 g, 16.6 mmol), the final purification by flash column chromatography on silica gel yielded **27b** (2.11 g, 14.6 mmol, 88%, *dr* > 95:5 by GC/MS analysis) as colourless oil.

TLC (hexane/ethyl acetate = 1:1): R_f = 0.47. GC (BPX-5): *I* = 1106. ^{1}H NMR (400 MHz, $CDCl_3$, TMS): δ = 3.67 (dd, $^3J_{H,H}$ = 4.7 Hz, $^2J_{H,H}$ = 12.2 Hz, 1H, CH_2), 3.56 (dd, $^3J_{H,H}$ = 7.7 Hz, $^2J_{H,H}$ = 12.4 Hz, 1H, CH_2), 2.71 (d, $^3J_{H,H}$ = 9.1 Hz, 1H, CH), 2.00 (m, 1H, OH), 1.44-1.22 (m, 3H, CH, CH_2), 1.28 (s, 3H, CH_3), 1.06 (d, $^3J_{H,H}$ = 6.3 Hz, 3H, CH_3), 0.89 (t, $^3J_{H,H}$ = 7.3 Hz, 3H, CH_3) ppm. ^{13}C NMR (100 MHz, $CDCl_3$, TMS): δ = 65.5 (CH_2), 65.4 (CH), 61.9 (C_q), 33.7 (CH), 26.7 (CH_2), 17.6 (CH_3), 14.8 (CH_3), 11.5 (CH_3) ppm. IR (ATR) $\tilde{\nu}$ = 3424 (m), 2962 (m), 2930 (m), 2875 (w), 1751

(w), 1461 (m), 1383 (m), 1241 (w), 1213 (w), 1073 (w), 1034 (s), 926 (w), 888 (s), 810 (m), 770 (w), 696 (m), 586 (m) cm^{-1}. UV-Vis (CH_2Cl_2): λ_{max} (log ε) = 230 (2.27), 222 (2.10) nm. MS (EI, 70 eV): *m*/*z* (%) = 126 (1) $[M-H_2O]^+$, 111 (2), 97 (2), 87 (5), 75 (8), 74 (6), 71 (9), 70 (8), 69 (27), 67 (7), 59 (15), 58 (36), 57 (57), 56 (7), 55 (48), 53 (15), 51 (8), 50 (6), 45 (25), 43 (91), 42 (21), 40 (14), 39 (100), 38 (12), 37 (3). Optical rotation: $[\alpha]_D^{20}$ = +20.0 (*c* 0.1, ethanol).

(4*S*,5*S*,6*S*)-5-((*tert*-Butyldimethylsilyl)oxy)-4,6-dimethyloctan-3-ol (29a)

To a solution of the epoxide **27a** (2.11 g, 14.60 mmol), 4 Å molecular sieves (2.92 g), and then diisopropylethylamine (2.54 g, 19.7 mmol) and *tert*-butyldimethylsilyl trifluoromethanesulfonate (5.02 g, 19.0 mmol) in anhydrous dichloromethane (44 mL) were added dropwise at −42 °C, followed by stirring for 2 hours at this temperature. The mixture was poured on phosphate buffer (100 mL, pH 5.5) and extracted three times with diethyl ether. The collected organic layers were washed with distilled water, 5% $NaHCO_3$ and brine, dried over $MgSO_4$ and concentrated in vacuo to obtain a colourless oil that was used for the next step without further purification.

The obtained crude aldehyde **28a** was dissolved in anhydrous diethyl ether (40 mL), and freshly prepared EtMgBr (38.2 mL, 38.2 mmol, 1.0

mol/L) was added dropwise at 0 °C. The mixture was stirred for 2 h at this temperature, followed by hydrolysis with distilled water. The aqueous layer was extracted three times with diethyl ether, the combined extracts were dried over $MgSO_4$ and concentrated in vacuo. The crude product was purified by flash column chromatography on silica gel to obtain **29a** (2.03 g, 7.00 mmol, 48%, colourless oil) as a mixture of diastereomers (*dr* = 55:45).

TLC (hexane/ethyl acetate = 20:1): R_f = 0.21. GC (HP-5): *I* = 1615 (major diastereomer) and *I* = 1594 (minor diastereomer). ^{1}H NMR (400 MHz, $CDCl_3$, TMS): δ = 3.72 (dd, $^3J_{H,H}$ = 3.0 Hz, $^3J_{H,H}$ = 4.7 Hz, 1H, CH), 3.52 (ddd, $^3J_{H,H}$ = 3.4 Hz, $^3J_{H,H}$ = 6.2 Hz, $^3J_{H,H}$ = 7.0 Hz, 1H, CH), 2.01 (br s, 1H, OH), 1.71-1.53 and 1.18-1.04 (m, 6H, 2xCH, 2xCH_2), 0.99-0.80 (m, 21H, 7xCH_3), 0.11-0.05 (m, 6H, 2xCH_3) ppm. ^{13}C NMR (100 MHz, $CDCl_3$, TMS, major diastereomer): δ = 79.2 (CH), 77.4 (CH), 40.8 (CH), 38.7 (CH), 27.7 (CH_2), 26.1 (3xCH_3), 25.9 (CH_2), 18.4 (C_q), 15.2 (CH_3), 12.3 (CH_3), 10.5 (CH_3), 8.4 (CH_3), –3.4 (CH_3), –4.4 (CH_3) ppm. IR (ATR): $\tilde{\nu}$ = 3462 (w), 2959 (m), 2932 (m), 2880 (w), 2858 (w), 1463 (w), 1382 (w), 1253 (m), 1109 (w), 1045 (m), 1002 (m), 971 (m), 833 (s), 772 (s), 675 (m) cm^{-1}. UV-Vis (CH_2Cl_2): λ_{max} (log ε) = 286 (2.54), 232 (4.35) nm. Optical rotation: $[\alpha]_D^{20}$ = +12.9 (*c* 0.1, ethanol). MS (EI, 70 eV, major diastereomer): *m*/*z* (%) = 231 (17) $[M-C(CH_3)_3]^+$, 230 (7), 213 (6), 202 (12), 201 (57), 175 (8), 174 (15), 173 (100), 162 (5), 161 (42), 147 (20),

145 (11), 143 (11), 133 (13), 119 (9), 115 (32), 105 (18), 83 (10), 77 (6), 76 (7), 75 (91), 74 (6), 73 (58), 69 (14), 59 (8), 57 (10), 55 (8), 43 (10), 41 (9).

(4*R*,5*R*,6*S*)-5-((*tert*-Butyldimethylsilyl)oxy)-4,6-dimethyloctan-3-ol (29b)

The same two-step procedure as for the preparation of **29a** was used. The starting material **27a** (1.31 g, 9.10 mmol) was converted into the target compound **29a** (0.70 g, 2.43 mmol, 27%) as a mixture of diastereomers (*dr* = 2:1) that was obtained as a colourless oil.

TLC (hexane/ethyl acetate = 10:1): R_f = 0.35. GC (HP-5MS): *I* = 1628 (major diastereomer) and *I* = 1607 (minor diastereomer). ^{1}H NMR (400 MHz, $CDCl_3$, TMS): δ = 3.66 (dd, $^{3}J_{H,H}$ = 3.9 Hz, $^{3}J_{H,H}$ = 3.9 Hz, 1H, CH), 3.54 (ddd, $^{3}J_{H,H}$ = 3.2 Hz, $^{3}J_{H,H}$ = 6.1 Hz, $^{3}J_{H,H}$ = 7.0 Hz, 1H, CH), 1.77 (s, 1H, OH), 1.69-1.61 (m, 1H, CH), 1.61-1.51 (m, 2H, CH_2, CH), 1.51-1.43 (m, 2H, CH_2), 1.17-1.05 (m, 1H, CH_2), 0.93 (t, $^{3}J_{H,H}$ = 7.5 Hz, 3H, CH_3), 0.91 (s, 9H, 3xCH_3), 0.89 (d, $^{3}J_{H,H}$ = 7.0 Hz, 3H, CH_3), 0.88 (t, $^{3}J_{H,H}$ = 7.3 Hz, 3H, CH_3), 0.86 (d, $^{3}J_{H,H}$ = 6.9 Hz, 3H, CH_3), 0.08 (s, 3H, CH_3), 0.07 (s, 3H, CH_3) ppm. ^{13}C NMR (100 MHz, $CDCl_3$, TMS): δ = 79.0 (CH), 76.3 (CH), 40.2 (CH), 39.7 (CH), 28.1 (CH_2), 26.1 (3xCH_3), 25.8 (CH_2), 18.4 (C_q), 14.8 (CH_3), 12.5 (CH_3), 10.5 (CH_3), 8.7 (CH_3), –3.4 (CH_3), –4.2 (CH_3) ppm. IR (ATR): $\tilde{\nu}$ = 3450 (w), 2959 (m), 2932 (m), 2880 (w), 2859

(w), 1463 (m), 1383 (w), 1253 (m), 1047 (m), 1002 (m), 968 (m), 833 (s), 772 (s), 674 (m) cm^{-1}. UV-Vis (CH_2Cl_2): λ_{max} (log ε) = 242 (2.95) nm. MS (EI, 70 eV, major diastereomer): *m*/*z* (%) = 231 (12) $[M-C(CH_3)_3]^+$, 202 (10), 201 (60), 175 (7), 174 (13), 173 (84), 161 (37), 147 (23), 145 (13), 143 (8), 133 (13), 119 (25), 117 (9), 116 (29), 106 (10), 105 (11), 94 (5), 84 (10), 77 (6), 76 (7), 75 (100), 74 (6), 73 (65), 69 (19), 59 (10), 57 (11), 55 (10), 41 (9). Optical rotation: $[\alpha]_D^{20}$ = +2.5 (*c* 0.1, ethanol).

(4*R*,5*S*,6*S*)-5-((*tert*-Butyldimethylsilyl)oxy)-4,6-dimethyloctan-3-one (30a)

To a solution of the alcohol **29a** (1.32 g, 4.56 mmol) in anhydrous dichloromethane (9.0 mL), pyridinium chlorochromate (1.32 g, 9.12 mmol) was added and the mixture was stirred overnight at room temperature. The reaction mixture was filtered over silica gel, concentrated in vacuo and the residue was purified by flash column chromatography on silica gel to yield **30a** as a colourless oil (1.16 g, 4.03 mmol, 88%).

TLC (hexane/ethyl acetate = 10:1): R_f = 0.55. GC (HP-5MS): *I* = 1581. ^{1}H NMR (400 MHz, $CDCl_3$, TMS): δ = 3.88 (dd, $^3J_{H,H}$ = 4.0 Hz, $^3J_{H,H}$ = 5.8 Hz, 1H, CH), 2.69 (dq, $^3J_{H,H}$ = 7.0 Hz, $^3J_{H,H}$ = 5.9 Hz, 1H, CH), 2.56-2.41 (m, 2H, CH_2), 1.46-1.34 (m, 2H, CH, CH_2), 1.07 (d, $^3J_{H,H}$ = 7.0 Hz, 3H, CH_3), 1.03 (t, $^3J_{H,H}$ = 7.3 Hz, 3H, CH_3), 1.08-0.95 (m, 1H, CH_2), 0.90-0.83

(m, 15H, 5xCH_3), 0.05 (s, 3H, CH_3), 0.01 (s, 3H, CH_3) ppm. ^{13}C NMR (100 MHz, $CDCl_3$, TMS): δ = 214.2 (C_q), 76.4 (CH), 49.2 (CH), 40.7 (CH), 35.4 (CH_2), 26.1 (3xCH_3), 25.0 (CH_2), 18.4 (C_q), 15.6 (CH_3), 13.6 (CH_3), 12.3 (CH_3), 7.8 (CH_3), –4.1 (CH_3), –4.2 (CH_3) ppm. IR (ATR): $\tilde{\nu}$ = 2959 (m), 2933 (m), 2880 (w), 2858 (w), 1713 (m), 1674 (w), 1461 (m), 1380 (w), 1254 (m), 1099 (m), 1051 (m), 1006 (m), 973 (m), 866 (m), 834 (s), 803 (m), 773 (s), 773 (m) cm^{-1}. UV-Vis (CH_2Cl_2): λ_{max} (log ε) = 237 (2.40) nm. MS (EI, 70 eV): *m/z* (%) = 286 (1) $[M]^+$, 230 (18), 229 (100), 206 (16), 201 (13), 173 (10), 145 (26), 143 (76), 115 (16), 75 (65), 73 (24), 56 (24), 44 (16), 40 (13). Optical rotation: $[\alpha]_D^{20}$ = –10.8 (*c* 0.37, ethanol).

(4*S*,5*R*,6*S*)-5-((*tert*-Butyldimethylsilyl)oxy)-4,6-dimethyloctan-3-one (30b)

The same procedure as for **30a** was used. The starting material **29b** (381 mg, 1.32 mmol) was converted into **30b** (328 mg, 1.14 mmol, 86%) that was obtained as colourless oil.

TLC (hexane/ethyl acetate = 10:1): R_f = 0.55. GC (HP-5MS): *I* = 1591. ^{1}H NMR (400 MHz, $CDCl_3$, TMS): δ = 3.85 (dd, $^3J_{H,H}$ = 2.9 Hz, $^3J_{H,H}$ = 6.7 Hz, 1H, CH), 2.73 (dq, $^3J_{H,H}$ = 7.0 Hz, $^3J_{H,H}$ = 7.0 Hz, 1H, CH), 2.52 (dq, $^2J_{H,H}$ = 18.0 Hz, $^3J_{H,H}$ = 7.2 Hz, 1H, CH_2), 2.45 (dq, $^2J_{H,H}$ = 18.0 Hz, $^3J_{H,H}$ = 7.2 Hz, 1H, CH_2), 1.49-1.37 (m, 1H, CH_2), 1.36-1.32 (m, 1H, CH), 1.19-1.07 (m, 1H, CH_2), 1.07 (d, $^3J_{H,H}$ = 7.2 Hz, 3H, CH_3), 1.03 (t, $^3J_{H,H}$ = 7.3

Hz, 3H, CH_3), 0.89 (s, 9H, 3xCH_3), 0.86 (t, $^3J_{H,H}$ = 7.4 Hz, 3H, CH_3), 0.80 (d, $^3J_{H,H}$ = 6.9 Hz, 3H, CH_3), 0.05 (s, 3H, CH_3), 0.04 (s, 3H, CH_3) ppm. ^{13}C NMR (100 MHz, $CDCl_3$, TMS): δ = 214.4 (C_q), 76.4 (CH), 50.2 (CH), 39.8 (CH), 35.8 (CH_2), 26.8 (CH_2), 26.1 (3xCH_3), 18.4 (C), 14.3 (CH_3), 14.0 (CH_3), 12.2 (CH_3), 7.7 (CH_3), –3.8 (CH_3), –4.0 (CH_3) ppm. IR (ATR): $\tilde{\nu}$ = 2960 (m), 2932 (m), 2881 (w), 2858 (w), 1712 (m), 1674 (w), 1461 (m), 1381 (w), 1253 (m), 1099 (m), 1051 (m), 1007 (m), 973 (m), 834 (s), 803 (m), 772 (s), 673 (m) cm^{-1}. UV-Vis (CH_2Cl_2): λ_{max} (log ε) = 287 (2.56), 232 (4.29) nm. MS (EI, 70 eV): *m*/*z* (%) = 271 (1) $[M-CH_3]^+$, 230 (17), 229 (100), 201 (12), 173 (10), 145 (14), 143 (76), 115 (13), 75 (52), 73 (21), 57 (21). Optical rotation: $[\alpha]_D^{20}$ = +13.2 (*c* 0.36, ethanol).

(4*R*,5*S*,6*S*)-5-Hydroxy-4,6-dimethyloctan-3-one (11c)

To a solution of **30a** (178 mg, 0.62 mmol) in anhydrous THF (5 mL), HF-pyridine (5.2 mL, 70% HF) was added carefully at 0 °C and the mixture was stirred at this temperature for 1 h. The reaction was quenched with aqueous $NaHCO_3$ solution until neutral pH, followed by threefold extraction with diethyl ether. The collected organic layers were dried over $MgSO_4$ and concentrated in vacuo. The residue was purified by column chromatography on silica gel to yield **11c** (48 mg, 0.31 mmol, 50%) as colourless oil.

TLC (hexane/ethyl acetate = 5:1): R_f = 0.33. GC (HP-5MS): *I* = 1240. ^{1}H NMR (400 MHz, $CDCl_3$, TMS): δ = 3.62 (dt, $^{4}J_{H,H}$ = 2.6 Hz, $^{3}J_{H,H}$ = 8.9 Hz, 1H, CH), 2.88 (d, $^{4}J_{H,H}$ = 3.0 Hz, 1H, OH), 2.74 (dq, $^{4}J_{H,H}$ = 2.5 Hz, $^{3}J_{H,H}$ = 7.2 Hz, 1H, CH), 2.63-2.44 (m, 2H, CH_2), 1.84-1.3 (m, 2H, CH_2), 1.20-1.14 (m, 1H, CH), 1.11 (d, $^{3}J_{H,H}$ = 7.2 Hz, 3H, CH_3), 1.07 (t, $^{3}J_{H,H}$ = 7.2 Hz, 3H, CH_3), 0.91(t, $^{3}J_{H,H}$ = 7.4 Hz, 3H, CH_3), 0.81 (d, $^{3}J_{H,H}$ = 6.8 Hz, 3H, CH_3) ppm. ^{13}C NMR (100 MHz, $CDCl_3$, TMS): δ = 217.1 (C_q), 74.4 (CH), 47.0 (CH), 36.8 (CH), 34.8 (CH_2), 25.0 (CH_2), 14.8 (CH_3), 10.8 (CH_3), 9.1 (CH_3), 7.7 (CH_3) ppm. IR (ATR): $\tilde{\nu}$ = 3490 (w), 2996 (m), 2937 (m), 2878 (m), 1701 (s), 1459 (m), 1410 (w), 1379 (m), 1299 (w), 1237 (w), 1149 (w), 1105 (w), 967 (m), 846 (w), 8023 (w), 775 (w) cm^{-1}. UV-Vis (CH_2Cl_2): λ_{max} (log ε) = 229 (3.26), 201 (3.74) nm. MS (EI, 70 eV): *m*/*z* (%) = 154 (6) $[M-H_2O]^+$, 125 (9), 115 (28), 98 (4), 97 (23), 87 (14), 86 (68), 85 (5), 83 (4), 71 (5), 70 (6), 69 (29), 58 (11), 57 (100), 56 (5), 55 (9), 45 (13), 43 (7), 41 (15), 39 (3). Optical rotation: $[\alpha]_D^{20}$ = –3.3 (*c* 0.3, ethanol).

(4*S*,5*R*,6*S*)-5-Hydroxy-4,6-dimethyloctan-3-one (11d)

The same procedure as for **11c** was used. The ketone **30b** (162 mg, 0.57 mmol) was converted into **11d** (71 mg, 0.41 mmol, 72%) that was obtained as a colourless oil.

TLC (hexane/ethyl acetate = 5:1): R_f = 0.33. GC (HP-5MS): *I* = 1242. ^{1}H NMR (500 MHz, C_6D_6): δ = 3.57 (dd, $^{4}J_{H,H}$ = 4.7 Hz, $^{3}J_{H,H}$ = 6.5 Hz, 1H,

CH), 2.40 (dq, $^3J_{H,H}$ = 4.7 Hz, $^3J_{H,H}$ = 7.1 Hz, 1H, CH), 2.24 (s, 1H, OH), 2.04 (dq, $^2J_{H,H}$ = 18.0 Hz, $^3J_{H,H}$ = 7.2 Hz, 1H, CH_2), 1.96 (dq, $^2J_{H,H}$ = 18.0 Hz, $^3J_{H,H}$ = 7.2 Hz, 1H, CH_2), 1.40-1.30 (m, 1H, CH), 1.27-1.17 (m, 1H, CH_2), 0.99-0.90 (m, 1H, CH_2), 0.97 (d, $^3J_{H,H}$ = 6.7 Hz, 3H, CH_3), 0.96 (d, $^3J_{H,H}$ = 7.1 Hz, 3H, CH_3), 0.90 (t, $^3J_{H,H}$ = 7.2 Hz, 3H, CH_3), 0.79 (t, $^3J_{H,H}$ = 7.4 Hz, 3H, CH_3) ppm. ^{13}C NMR (125 MHz, C_6D_6): δ = 214.5 (C_q), 74.7 (CH), 48.2 (CH), 37.5 (CH), 34.9 (CH_2), 26.2 (CH_2), 14.5 (CH_3), 11.4 (CH_3), 11.2 (CH_3), 7.8 (CH_3) ppm. IR (ATR): $\tilde{\nu}$ = 3483 (w), 2966 (w), 2936 (w), 2878 (w), 1702 (s), 1459 (m), 1410 (w), 1378 (m), 1237 (w), 1148 (w), 1103 (m), 974 (m), 805 (w), 773 (w), 540 (m) cm^{-1}. UV-Vis (CH_2Cl_2): λ_{max} (log ε) = 224 (3.68), 202 (4.06) nm. MS (EI, 70 eV): *m/z* (%) = 154 (6) $[M-H_2O]^+$, 125 (8), 115 (22), 98 (3), 97 (20), 87 (11), 86 (56), 85 (4), 83 (3), 71 (5), 70 (4), 69 (22), 58 (9), 57 (100), 56 (4), 55 (7), 45 (11), 43 (6), 41 (12). Optical rotation: $[\alpha]_D^{20}$ = +1.8 (*c* 0.7, ethanol).

Epimerisation of (4*R*,5*S*,6*S*)-11c and (4*S*,5*R*,6*S*)-11d

To a solution of the ketone (4*R*,5*S*,6*S*)-**11c** or (4*S*,5*R*,6*S*)-**11d** (10 mg, 0.058 mmol) in methanol (0.6 mL), potassium carbonate (17 mg, 0.12 mmol) was added and the mixture was stirred at room temperature overnight. Distilled water (10 mL) was added and the aqueous phase was extracted extracted three times with ethyl acetate. The combined organic layers were dried over $MgSO_4$ and concentrated in vacuo. A

small sample of the epimerisation product was added to the mixture of eight stereoisomers of **11** in a ca. 1:5 ratio, followed by GC–MS analysis.

Chromatographic separation of stereoisomers of 11

A portion of the mixture of stereoisomers (100 mg) was subjected to purification. The racemate of **11d** was separated from the other six stereoisomers by column chromatography on silica gel, yielding (*rac*)-**11d** (30 mg). Subsequently, a sample of (*rac*)-**11d** (6.0 mg) was subjected to separation of the enantiomers by HPLC on a chiral stationary phase (DAICEL Chiralpak IA) to give (4*S*,5*R*,6*S*)-**11d** (3.0 mg) and (4*R*,5*S*,6*R*)-**11d** (3.0 mg).

The mixture of stereoisomers **11a**, **11b** and **11c** (70 mg) was separated by reversed phase HPLC using a KNAUER Europher II 100-5 C18 column to yield (*rac*)-**11a** (12 mg), (*rac*)-**11b** (15 mg) and (*rac*)-**11c** (20 mg). Separation of the racemates of **11a** and **11b** on a chiral stationary phase (DAICEL Chiralpak IA) finally gave pure (4*R*,5*R*,6*S*)-**11a** (6 mg) and (4*S*,5*S*,6*R*)-**11a** (6 mg), and (4*S*,5*S*,6*S*)-**11b** (7 mg) and (4*R*,5*R*,6*R*)-**11b** (7 mg). A separation of the enantiomers of **11c** via HPLC on a chiral column was not successful.

(4*R*,5*R*,6*S*)-5-Hydroxy-4,6-dimethyloctan-3-one (11a)

GC (HP-5MS): *I* = 1281. ^{1}H NMR (500 MHz, C_6D_6): δ = 3.62 (dd, $^3J_{H,H}$ = 2.4 Hz, $^3J_{H,H}$ = 8.3 Hz, 1H, CH), 2.46 (dq, $^3J_{H,H}$ = 7.2, $^3J_{H,H}$ = 8.3 Hz, 1H, CH), 2.13 (dq, $^2J_{H,H}$ = 18.1 Hz, $^3J_{H,H}$ = 7.3 Hz, 1H, CH_2), 2.09 (dq, $^2J_{H,H}$ = 18.1 Hz, $^3J_{H,H}$ = 7.2 Hz, 1H, CH_2), 1.36-1.22 (m, 2H, CH_2, CH), 1.22-1.12 (m, 1H, CH_2), 0.96 (t, $^3J_{H,H}$ = 7.3 Hz, 3H, CH_3), 0.83 (d, $^3J_{H,H}$ = 6.7 Hz, 3H, CH_3), 0.82 (t, $^3J_{H,H}$ = 7.3 Hz, 3H, CH_3), 0.75 (d, $^3J_{H,H}$ = 7.2 Hz, 3H, CH_3) ppm. ^{13}C NMR (125 MHz, C_6D_6): δ = 215.0 (C_q), 75.8 (CH), 48.8 (CH), 37.0 (CH), 36.1 (CH_2), 27.1 (CH_2), 13.9 (CH_3), 12.4 (CH_3), 12.0 (CH_3), 7.7 (CH_3) ppm. IR (ATR): $\tilde{\nu}$ = 3479 (w), 2963 (w), 2934 (w), 2877 (w), 1739 (w), 1702 (s), 1458 (m), 1409 (w), 1376 (m), 1299 (w), 1259 (w), 1233 (w), 1136 (w), 1106 (w), 1085 (w), 1021 (m), 972 (m), 956 (m), 867 (m), 799 (m), 736 (m), 702 (w), 516 (w), 446 (w) cm^{-1}. UV-Vis (CH_2Cl_2): λ_{max} (log ε) = 231 (2.99) nm. MS (EI, 70 eV): *m*/*z* (%) = 154 (4) $[M-H_2O]^+$, 125 (9), 116 (4), 115 (57), 113 (5), 98 (6), 97 (24), 87 (9), 86 (46), 85 (4), 83 (6), 71 (4), 70 (8), 69 (35), 58 (8), 57 (100), 56 (5), 55 (8), 45 (7), 43 (5), 41 (11). Optical rotation: $[\alpha]_D^{20}$ = –2.4 (*c* 0.3, ethanol).

(4*S*,5*S*,6*R*)-5-Hydroxy-4,6-dimethyloctan-3-one (11a)

Analytical data were identical to those of (4*S*,5*R*,6*S*)-**11a**. Optical rotation: $[\alpha]_D^{20}$ = +1.3 (*c* 0.3, ethanol).

(4*S*,5*S*,6*S*)-5-Hydroxy-4,6-dimethyloctan-3-one (11b)

GC (HP-5MS): *I* = 1277. ^{1}H NMR (500 MHz, C_6D_6): δ = 3.35 (q, $^{3}J_{H,H}$ = 5.2 Hz, 1H, CH), 2.52 (br d, $^{3}J_{H,H}$ = 6.5 Hz, 1H, OH), 2.47 (dq, $^{3}J_{H,H}$ = 6.5, $^{3}J_{H,H}$ = 7.2 Hz, 1H, CH), 2.10 (dq, $^{2}J_{H,H}$ = 18.1 Hz, $^{3}J_{H,H}$ = 7.3 Hz, 1H, CH_2), 2.04 (dq, $^{2}J_{H,H}$ = 18.1 Hz, $^{3}J_{H,H}$ = 7.2 Hz, 1H, CH_2), 1.58 (ddq, $^{2}J_{H,H}$ = 13.4 Hz, $^{3}J_{H,H}$ = 7.6 Hz, $^{3}J_{H,H}$ = 3.2 Hz, 1H, CH_2), 1.39-1.29 (m, 1H, CH), 1.20 (ddq, $^{2}J_{H,H}$ = 13.4 Hz, $^{3}J_{H,H}$ = 7.4 Hz, $^{3}J_{H,H}$ = 9.5 Hz, 1H, CH_2), 0.93 (t, $^{3}J_{H,H}$ = 7.2 Hz, 3H, CH_3), 0.86 (d, $^{3}J_{H,H}$ = 7.2 Hz, 3H, CH_3), 0.85 (t, $^{3}J_{H,H}$ = 7.5 Hz, 3H, CH_3), 0.77 (d, $^{3}J_{H,H}$ = 6.8 Hz, 3H, CH_3) ppm. ^{13}C NMR (125 MHz, C_6D_6): δ = 215.6 (C), 78.4 (CH), 47.7 (CH), 38.1 (CH), 36.2 (CH_2), 23.6 (CH_2), 16.3 (CH_3), 14.7 (CH_3), 11.8 (CH_3), 7.7 (CH_3) ppm. IR (ATR): $\tilde{\nu}$ = 3459 (w), 2962 (w), 2935 (w), 2877 (w), 1710 (m), 1458 (m), 1411 (w), 1377 (m), 1258 (s), 1082 (m), 1010 (s), 864 (m), 790 (s), 734 (w), 700 (w), 661 (w), 407 (w) cm^{-1}. UV-Vis (CH_2Cl_2): λ_{max} (log ε) = 229 (3.39) nm. MS (EI, 70 eV): *m*/*z* (%) = 154 (4) $[M-H_2O]^+$, 125 (9), 116 (4), 115 (62), 114 (5), 98 (5), 97 (22), 87 (8), 86 (34), 85 (4), 83 (5), 71 (4), 70 (6), 69 (31), 58 (8), 57 (100), 56 (4), 55 (8), 45 (6), 43 (4), 41 (10). Optical rotation: $[\alpha]_D^{20}$ = +0.9 (*c* 0.2, ethanol).

(4*R*,5*R*,6*R*)-5-Hydroxy-4,6-dimethyloctan-3-one (11b)

Analytical data were identical to those of (4*R*,5*S*,6*S*)-**11b**. Optical rotation: $[\alpha]_D^{20}$ = –1.3 (*c* 0.3, ethanol).

(4*RS*,5*SR*,6*SR*)-5-Hydroxy-4,6-dimethyloctan-3-one (11c)

Analytical data of (*rac*)-**11c** were identical to those of (4*R*,5*S*,6*S*)-**11c** obtained by enantioselective synthesis.

(4*S*,5*R*,6*S*)-5-Hydroxy-4,6-dimethyloctan-3-one (11d)

Analytical data were identical to those of (4*S*,5*R*,6*S*)-**11d** obtained by enantioselective synthesis.

(4*R*,5*S*,6*R*)-5-Hydroxy-4,6-dimethyloctan-3-one (11d)

Analytical data were identical to those of (4*S*,5*R*,6*S*)-**11d**. Optical rotation: $[\alpha]_D^{20}$ = –1.7 (*c* 0.7, ethanol).

Pent-4-en-2-yl acrylate (33)

To a solution of pent-4-en-2-ol (**31**, 345 mg, 4.00 mmol) and triethylamine (559 mg, 5.52 mmol) in anhydrous dichloromethane (13.3 mL), acryloyl chloride (**32**, 362 mg, 4.00 mmol) was added dropwise at 0 °C, followed by stirring at this temperature for 1 h. Then, the reaction mixture was warmed to room temperature and stirred overnight. The reaction was quenched with 1 N HCl and extracted three times with dichloromethane. The collected organic phases were washed with saturated aqueous $NaHCO_3$ and brine, dried over $MgSO_4$ and concentrated in vacuo. Purification by column chromatography on silica gel afforded **33** (367 mg, 2.62 mmol, 66%) as pale yellow oil. The compound proved to be sensitive to slow spontaneous degradation.

TLC (hexane/ethyl acetate = 40:1): R_f = 0.2. GC (BPX-5): I = 922. ^{1}H NMR (400 MHz, $CDCl_3$, TMS): δ = 6.37 (dd, $^2J_{H,H}$ = 1.5 Hz, $^3J_{H,H}$ = 17.3 Hz, 1H, CH_2), 6.09 (dd, $^3J_{H,H}$ = 10.4, $^3J_{H,H}$ = 17.3 Hz, 1H, CH), 5.79 (dd, $^4J_{H,H}$ = 1.5 Hz, $^3J_{H,H}$ = 10.4 Hz, 1H, CH_2), 5.82-5.70 (m, 1H, CH), 5.11-5.04 (m, 2H, CH_2), 5.03 (sext, $^3J_{H,H}$ = 6.3 Hz, 1H, CH), 2.42-2.26 (m, 2H, CH_2), 1.25 (d, $^3J_{H,H}$ = 6.3 Hz, 3H, CH_3) ppm. ^{13}C NMR (100 MHz, $CDCl_3$, TMS): δ = 165.7 (C_q), 133.6 (CH_2), 130.3 (CH), 128.9 (CH_2), 117.7 (CH), 70.3 (CH), 40.2 (CH_2), 19.4 (CH_3) ppm. IR (ATR): $\tilde{\nu}$ = 3079 (w), 2978 (w), 2930 (w), 2854 (w), 1713 (s), 2637 (w), 1506 (w), 1451 (w), 1406 (w), 1381 (w), 1251 (m), 1192 (m), 1121 (m), 1051 (m), 991 (m), 920 (m), 801 (m), 587 (w) cm^{-1}. UV-Vis (CH_2Cl_2): λ_{max} (log ε) = 251 (4.25), 226 (4.22), 222 (4.17) nm. MS (EI, 70 eV): m/z (%) = 140 (0.2) $[M]^+$, 100 (1), 99 (24), 96 (2), 69 (4), 68 (8), 67 (6), 56 (4), 55 (100), 53 (5), 45 (3), 43 (7), 41 (20), 39 (18).

6-Methyl-5,6-dihydro-*2H*-pyran-2-one (9)

To a solution of **33** (290 mg, 2.07 mmol) in anhydrous dichloromethane (40 mL), Hoveyda–Grubbs II catalyst (13 mg, 0.021 mmol) was added and the mixture was stirred under reflux for 4 h. The reaction was cooled to room temperature and concentrated in vacuo. The residue was purified by flash column chromatography on silica gel to yield the title compound **9** (60 mg, 0.54 mmol, 26%) as pale yellow oil.

TLC (hexane/ethyl acetate = 1:1): R_f = 0.20. GC (HP-5): *I* = 1074. (BPX-5): *I* = 1100. ^{1}H NMR (400 MHz, $CDCl_3$, TMS): δ = 6.86 (ddd, $^3J_{H,H}$ = 8.5 Hz, $^3J_{H,H}$ = 5.8 Hz, $^3J_{H,H}$ = 2.7 Hz, 1H, CH), 6.00 (m, 1H, CH), 4.56 (ddq, $^3J_{H,H}$ = 4.5 Hz, $^3J_{H,H}$ = 10.8 Hz, $^3J_{H,H}$ = 6.4 Hz, 1H, CH), 2.36 (dddd, $^4J_{H,H}$ = 1.2 Hz, $^3J_{H,H}$ = 4.4 Hz, $^3J_{H,H}$ = 5.6 Hz, $^2J_{H,H}$ = 18.2 Hz, 1H, CH_2), 2.28 (dddd, $^4J_{H,H}$ = 2.7 Hz, $^3J_{H,H}$ = 2.7 Hz, $^3J_{H,H}$ = 11.1 Hz, $^2J_{H,H}$ = 18.4 Hz, 1H, CH_2), 1.42 (d, $^3J_{H,H}$ = 6.3 Hz, 3H, CH_3) ppm. ^{13}C NMR (100 MHz, $CDCl_3$, TMS): δ = 164.5 (C_q), 144.9 (CH), 121.2 (CH), 74.3 (CH), 31.0 (CH_2), 20.7 (CH_3) ppm. IR (ATR): $\tilde{\nu}$ = 2981 (w), 2937 (w), 2906 (w), 1715 (s), 1389 (m), 1245 (s), 1167 (w), 1107 (m), 1051 (s), 995 (w), 925 (m), 886 (w), 848 (m), 813 (s), 700 (w), 662 (m) cm^{-1}. UV-Vis (CH_2Cl_2): λ_{max} (log ε) = 228 (4.08) nm. MS (EI, 70 eV): *m*/*z* (%) = 112 (3) $[M]^+$, 97 (13), 69 (19), 68 (100), 53 (5), 43 (16), 42 (15), 41 (20), 40 (32), 39 (45), 38 (9), 38 (4).

6-Nonyl-2*H*-pyran-2-one (17)

Propiolic acid (**35**, 49 mg, 0.70 mmol) was added to a solution of 1-undecyne (**34**, 106 mg, 0.70 mmol) and chloro(triphenylphosphine)gold(I) (4 mg, 0.01 mmol) in anhydrous dichloromethane (4 mL) in a sealed tube. Then, silver trifluoromethanesulfonate (2 mg, 0.01 mmol) was added and the tube was closed instantly. The mixture was heated to 50 °C for 15 h. The solvent was evaporated and the residue was purified

by column chromatography on silica gel to obtain 6-nonyl-2*H*-pyran-2-one (**17**, 46 mg, 0.12 mmol, 30%) as colourless oil.

TLC (hexane/ethyl acetate = 15:1): R_f = 0.12. GC (HP-5): *I* = 1889. (BPX-5): *I* = 1905. ^{1}H NMR (400 MHz, $CDCl_3$, TMS): δ = 7.24 (dd, $^3J_{H,H}$ = 6.5 Hz, $^3J_{H,H}$ = 9.4 Hz, 1H, CH), 6.14 (dd, $^4J_{H,H}$ = 0.5 Hz, $^3J_{H,H}$ = 9.4 Hz, 1H, CH), 5.96 (dd, $^4J_{H,H}$ = 0.8 Hz, $^3J_{H,H}$ = 6.5 Hz, 1H, CH), 2.47 (t, $^3J_{H,H}$ = 7.6 Hz, 2H, CH_2), 1.64 (quin, $^3J_{H,H}$ = 7.6 Hz, 2H, CH_2), 1.37-1.19 (m, 12H, 6xCH_2), 0.87 (t, $^3J_{H,H}$ = 6.8 Hz, 3H, CH_3) ppm. ^{13}C NMR (100 MHz, $CDCl_3$, TMS): δ = 166.8 (C_q), 162.9 (C_q), 143.7 (CH), 113.0 (CH), 102.6 (CH), 33.8 (CH_2), 31.8 (CH_2), 29.4 (CH_2), 29.2 (2xCH_2), 28.9 (CH_2), 26.8 (CH_2), 22.6 (CH_2), 14.1 (CH_3) ppm. IR (ATR): $\tilde{\nu}$ = 2923 (s), 2853 (m), 1726 (s), 1633 (m), 1557 (s), 1464 (m), 1397 (w), 1377 (w), 1354 (w), 1266 (w), 1172 (w), 1085 (m), 979 (w), 892 (w), 852 (w), 796 (s), 722 (m), 548 (w), 491 (w) cm^{-1}. UV-Vis (CH_2Cl_2): λ_{max} (log ε) = 300 (4.65), 217 (4.39) nm. MS (EI, 70 eV): *m*/*z* (%) = 222 (14) $[M]^+$, 137 (20), 123 (37), 110 (47), 96 (13), 95 (100), 94 (17), 82 (49), 81 (45), 69 (11), 67 (16), 55 (23), 53 (18), 43 (32), 41 (52), 39 (67).

Bioactivity testings

The biological activities of the synthetic compounds were evaluated using the methods described by Sudarman et al. [3]. Compound

solutions were prepared with acetone as solvent, pure acetone was used for negative controls.

References

[1] Stadler, M.; Læssøe, T.; Fournier, J.; Decock, C.; Schmieschek, B.; Tichy, H. V.; Peršoh, D. *Stud. Mycol.,* **2014,** *77,* 1-143.

[2] Pažoutová, S.; Follert, S.; Bitzer, J.; Keck, M.; Surup, F.; Šrůtka, P.; Holuša, J.; Stadler, M. *Fungal Divers.,* **2013,** *60,* 107-123.

[3] Sudarman, E.; Kuhnert, E.; Hyde, K. D.; Sir, E. B.; Surup, F.; Stadler, M. *Tetrahedron,* **2016,** *72,* 6450-6454.
